# Antibacterial, Photoprotective, Anti-Inflammatory, and Selected Anticancer Properties of Honokiol Extracted from Plants of the Genus *Magnolia* and Used in the Treatment of Dermatological Problems—A Review

**DOI:** 10.3390/ijms26178737

**Published:** 2025-09-08

**Authors:** Mirosława Chwil, Katarzyna Dzida, Paulina Terlecka, Daniela Gruľová, Renata Matraszek-Gawron, Karol Terlecki, Anna Kasprzyk, Mikołaj Kostryco

**Affiliations:** 1 Department of Botany and Plant Physiology, University of Life Sciences in Lublin, Akademicka 15 St., 20-950 Lublin, Poland; miroslawa.chwil@up.lublin.pl (M.C.); kostryco@gmail.com (M.K.); 2 Institute of Horticulture Production, Subdepartment of Plant Nutrition, University of Life Sciences in Lublin, Głęboka 28 St., 20-612 Lublin, Poland; katarzyna.dzida@up.lublin.pl; 3 Department of Endocrinology, Diabetology and Metabolic Diseases, Medical University of Lublin, Jaczewskiego 8 Street, 20-090 Lublin, Poland; paulinaterlecka@umlub.edu.pl; 4 Department of Ecology, University of Prešov in Prešov, 17th November 1 St., 080 01 Presov, Slovakia; daniela.grulova@unipo.sk; 5 Department of Vascular Surgery and Angiology, Medical University of Lublin, Aleja Solidarności 8 St., 20-081 Lublin, Poland; karol.terlecki@umlub.pl; 6 Department of Animal Breeding and Agricultural Consulting, University of Life Sciences in Lublin, Akademicka 13 St., 20-950 Lublin, Poland; anna.kasprzyk@up.lublin.pl

**Keywords:** anti-proliferative effect, biofilms, 3,5-di-2-propylene-1,1-biphenyl-2,4-diol, phytotherapy, antiviral and antibacterial effects, antifungal properties, anti-melanoma effects

## Abstract

*Magnolia* raw materials have long been used in Chinese folk medicine. The biologically active chemical compounds in *Magnolia*, mainly lignans, e.g., honokiol, exert health-enhancing effects in certain diseases, including skin conditions. Since the scientific literature does not provide a comparative analysis of the therapeutic properties of honokiol on the skin in various biological models, an attempt was made to supplement the knowledge in this field. This review presents the antimicrobial, anti-inflammatory, and photoprotective properties of honokiol used in dermatological problems and its anticancer activity in melanoma and non-melanoma skin cancers. Honokiol reduces the expression of HSV-1 genes, inhibits DNA replication, lowers the level of proteins, regulates the colonisation of viral glycoproteins with high membrane selectivity, and inhibits the endocytosis process. It has antibacterial activity, as it destroys bacterial cell walls and membranes. It disrupts vacuolar functioning and intracellular calcium homeostasis in dermatophyte cells and inhibits fungal growth by delaying germination, altering membrane permeability, and reducing hyphal growth. It reduces inflammatory cytokines and stimulates anti-inflammatory cytokine IL-10. Honokiol prevents UV-B induced skin cancer through targeting cell cycle regulators, inflammatory mediators, and cell survival signals. It induces apoptosis via extrinsic and intrinsic pathways, activating proapoptotic proteins. It acts as an inhibitor of the oncogenic protein KRT18 in melanoma and prevents the progression of highly metastatic melanoma. Future research should explore the signalling pathways and molecular mechanisms of honokiol action and its synergistic effects at the cellular level and help to develop methods for delivering honokiol to the organism by nanocarriers to improve selective therapies in some diseases.

## 1. Introduction

Currently, the use of phytotherapy in many diseases, including dermatological problems, is being intensively investigated [1,2,3]. Numerous experiments conducted in various biological models have evidenced that honokiol extracted from various raw materials of many species of the genus *Magnolia* [4,5] has promising potential as a component of phytotherapy for skin diseases, including those caused by microorganisms [2,6,7,8]. The health-enhancing properties of honokiol and its use in many dermatological applications have been documented [2,9,10,11,12,13,14]. This phytocompound may be a promising ingredient in many pharmacological and cosmetic formulations [11,15,16,17,18,19]. Additionally, honokiol can be applied in the treatment of oxidative stress-induced skin diseases, as it was shown to prevent oxidative damage via AMPK stimulation in human HaCaT keratinocytes. It reduced cytotoxicity, inhibited apoptosis, and limited hydrogen peroxide generation [20]. It decreased the calcium concentration in cytosol in NHEK and HEK293T cells with overexpression of hTRPV3 and inhibited the release of IL-6 and IL-8 via TRPV3 in keratinocytes, which indicates that it can be used in phytotherapy of inflammatory skin diseases [21].

It has been shown that honokiol inhibits fibroblast activity and behaviour in various conditions, including hypertrophic scars, epidural fibrosis, and oral fibrogenesis, which indicates its role as a potential healing agent for fibrotic diseases. Honokiol affects fibroblasts via inhibition of the overproduction of extracellular matrix components, downregulation of fibrosis-related molecules, repression of migration and proliferation, and activation of myofibroblasts via the Smad-dependent pathway [2,22,23].

Honokiol, a natural pleiotropic lignan, is widely used in traditional medicine. It has been defined as a novel, unique compound with multiple biological activities and effects on the living organism, influencing various catabolic and anabolic biological processes and potentially interacting with multiple targets or pathways [24]. All these actions make honokiol and its derivates serve as the lead ingredient in formulations with high efficacy and minor side effects applied in the treatment of several disorders [24,25].

New potential therapeutic applications for honokiol and its derivatives are being sought; however, their modes of action and potential to be used for development of secure and efficient treatments remain poorly understood and require further intensified research. Ongoing investigations are focused on acquisition of innovative honokiol compounds with increased biological activity, enhanced effects, and improved pharmacological characteristics by enriching the structure of this compound with various functional halogen, methyl, ethyl, or amino groups, i.e., electron-donating groups enhancing the antioxidant properties of this neolignan [24,26]. Hydroxyl (-OH) groups of honokiol and its derivatives raise its antioxidant and free-radical scavenging properties. The biphenolic structure of honokiol determining its biological function and interactions with target sites and receptors can be modified by changing the rings or adding fused ring systems; i.e., changes in aryl or alkyl groups in honokiol derivatives determine their lipophilicity and bioavailability, and replacing the honokiol propyl group with a more hydrophobic one enhances its solubility and bioavailability [24,26].

To the best of our knowledge, there are only few original research publications that synthetically summarise the information on selected biological activities of honokiol and its derivatives in terms of their use in the treatment of dermatological problems. Since the scientific literature does not provide a comparative analysis of the therapeutic properties of honokiol, e.g., its antimicrobial, anti-inflammatory, photoprotective, and anticancer effects on the skin in various biological models, an attempt was made to supplement the knowledge in this field. Recent data published after 2015 were included, with a special analysis of scientific reports from the last five years on the mechanisms of action of honokiol determining its activities.

The aim of the review was to present the therapeutic antiviral, antibacterial, antifungal, anti-inflammatory, and photoprotective properties of honokiol applied in the therapy for various dermatological conditions and its anti-cancer applications against melanoma and non-melanoma skin cancers, basalioma, and epidermoid carcinoma based on a review of the latest original research publications.

## 2. Systematics and Occurrence of *Magnolia* Genus Plants Serving as Honokiol Sources

Extracts of *Magnolia* species have long been used in traditional medicine in Korea, China, and Japan [27,28]. The genus *Magnolia* belongs to the subfamily Magnolioideae, family Magnoliaceae, order Magnoliales, and clade Magnoliids [29,30,31,32,33,34]. The clade Magnoliids comprises approximately 10,000 species classified into four orders and 18 families [34]. The order Magnoliales is composed of six families. The family Magnoliaceae comprises plants with different habits, vines, shrubs, and trees, representing 335 species classified into 18 genera [29,35]. The name of the family Magnoliaceae derives from the surname of French botanist Professor Pierre Magnol (17th/18th century) [36,37]. Another French botanist and specialist in systematic plant anatomy, Paul Évariste Parmentier (19th/20th century), presented a detailed history of the family Magnoliaceae [38]. The genus *Magnolia* comprises 370 species occurring in their natural habitat in Europe, Asia, and the eastern and southern regions of America [39]. Some species of the genus *Magnolia* acclimatise well to variable climatic conditions [40]. In Poland, the family Magnoliaceae is represented by various species from the genera *Magnolia* and *Liriodendron* cultivated for ornamental purposes [41,42,43,44,45].

## 3. Honokiol, Its Derivatives, and Other Active Compounds in *Magnolia* Extracts

**Figure 1:**
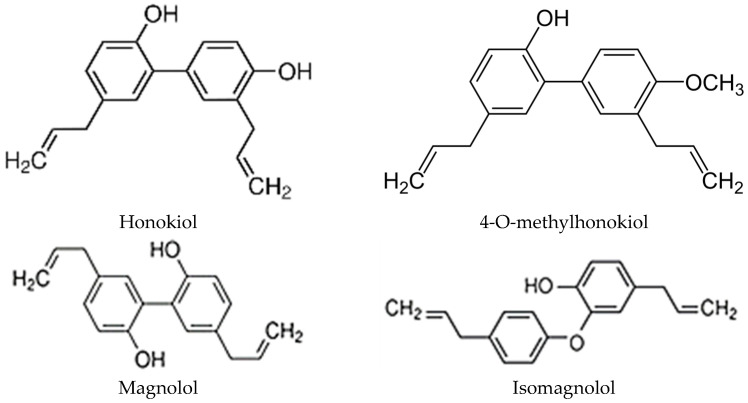
Structural formulas of honokiol, 4-O-methylhonokiol, magnolol, and isomagnolol.

For protection in the interactions with various environmental factors, plants synthesise biologically active chemical compounds [46]. It has been evidenced that active compounds representing various chemical classes and originating from various *Magnolia* species possess therapeutic properties. Ingested with the diet or administered via other routes, they activate certain biochemical pathways in metabolic processes occurring in the organism [47,48,49,50,51]. The phytochemical analysis of extracts from the bark and flowers of *Magnolia* species *M. champaca*, *M. denudata*, *M. grandiflora,* and *M. officinalis* showed high amounts of antioxidant polyphenols (55.18 mg·GAE g^−1^) in the chemical profile, with IC50 in the range of 9.99–23.23 mg·mL^−1^. The content of phenolic acids, glycoside, aglycone flavonoids, and lignans was within the range of (mg·g^−1^ D.M.) 6.26–27.88, 17.27–57.96, 61.22–135.79, and 150.07–374.90, respectively. Additionally, 76 vaporescent compounds, primarily monoterpenes and sesquiterpene hydrocarbons, responsible for the bacteriostatic and bactericidal effects of magnolia extracts, were identified [52]. The chemical profile of magnolia leaves comprised 20 different chemical compounds, including 16 lignans representing six distinct structural types, e.g., sitosterol, honokiol, magliflonenone, and veraguensin [5]. Lignans were represented (mg·g^−1^ D.M.) by 3-methoxymagnolol (1.29–7.00 bark/0.23–5.66 flower), 4-O-methylhonokiol (2.01–10.02/1.02–18.10), honokiol (5.09–56.79/2.10–18.92), isomagnolol (1.03–6.79/0.10–7.78), and magnolol (7.02–97.09/2.00–23.02). Extracts from the bark and flowers of *Magnolia* plants were dominated by honokiol and magnolol, with analogues of these compounds present in lower concentrations. The concentration of these compounds was significantly higher in the bark than in the flowers [52]. The structural formulas of these compounds are shown in Figure 1.

## 4. Methods of Honokiol Extraction

Honokiol and magnolol are the best-known active components from the over 250 substances extracted from the bark, seed cones, and leaves of plants of the genus *Magnolia*, mainly *Magnolia officinalis*, *Magnolia grandiflora*, and other *Magnolia* species, e.g., *Magnolia dealbata* [2]. Extracts from the bark and seed cones of *Magnolia* trees have been traditionally used in Chinese (Houpu formula), Japanese (Kampo formula), and Korean medicine as an analgesic, anxiolytic, and anti-stroke agent [11,53,54]. Honokiol is extracted in a process consisting of the following steps: fragmentation of the raw material, extraction using alkaline lye (aqueous hydroxide solution) or alcohol (methanol, ethanol), removal of impurities by adding acid, precipitation and purification with alcohol, chromatography, and refining; in some cases, honokiol may be further purified by acid precipitation and crystallisation [55,56]. Traditional extraction methods involve (1) alkali acid precipitation, where magnolia bark is extracted with a solvent (ethyl acetate C_4_H_8_O_2_); next, the extract is dissolved in an alkaline solution (KOH), and honokiol is precipitated by adding acid (CH_3_COOH) to adjust the pH; (2) solvent solution to extract bark with solvents like methanol, ethanol, or ethyl acetate, followed by further purification steps; and (3) chromatography (liquid chromatography) and crystallisation, used to further purify honokiol from raw extracts [56]. The modern extraction approaches involve mechanochemical extraction that utilises mechanical force to facilitate reactions between solid-phase reagents and the plant material, leading to the formation of water-soluble compounds. Noteworthy, nanomaterials (nanoemulsions) can be used to enhance honokiol solubility and therefore bioavailability [18,57].

As a highly hydrophobic natural bisphenol neolignan, honokiol is extracted exclusively in a mixture with magnolol. Due to their structural resemblance and natural occurrence as structural isomers, these two neolignans cannot be easily separated using standard column or thin layer chromatography. The purification necessitates the use of electromigration or special advanced chromatographic conditions, including magnolol acetonide protection followed by high-capacity, high-speed, counter-current chromatography or flash chromatography, which significantly increases costs and impedes adaptation to large-scale production. Both neolignans differ in the −OH (hydroxyl) group position, but this group in magnolol seems to form a protectable diol, which facilitates selective chemical modification and, in consequence, facile separation [58]. Modern methods devised in 2006 by Jack L. Arbiser and laboratory workers from Emory University in Atlanta (GA, USA) took advantage of the proximity of the phenolic hydroxyl groups in magnolol, which, contrary to honokiol, form a protectable diol with 2,2-dimethoxypropane in catalytic acidic conditions to generate magnolol acetonide. Honokiol may be readily separated from the protected magnolol acetonide with subsequent simple and economical purification flash chromatography over silica, giving 96.8% purity as determined by high-performance liquid chromatography (HPLC). The purity of honokiol can be raised to 99.8% by repeating the reaction in the same manner on the partially purified honokiol [58]. Another modern rapid separation approach was developed as well, and this process involves high-capacity, high-speed, counter-current chromatography (high-capacity HSCCC). This liquid–liquid technique utilises a rotating coil to achieve separation and purification of compounds from crude extracts without using a solid support. For example, a two-phase solvent system composed of light petroleum, ethyl acetate, methanol, and 1% acetic acid (5:5:7:3, v/v) or n-hexane–ethyl acetate–methanol–water (1:0.4:1:0.4, v/v), enabling high-speed and efficient high capacity separation and purification of large quantities of compound can be used. With this method, honokiol is separated and purified to above 98% purity with a high yield within an hour [59,60,61]. A simple, rapid, and precise method for the separation and determination of honokiol and magnolol in magnolia raw material was built by the combination of flow injection and free solution capillary electrophoresis [62].

The most recent reports demonstrate the possibility to use *Magnolia officinalis* residues after prior alkali treatment to release remaining honokiol and magnolol and then absorbing these neolignans with metal–organic frame material MIL-101(Cr). Additionally, ß-amyrin can be obtained by saccharification and fermentation. A total of 1000 g of honokiol, 8000 g of magnolol, and 7640 g of triterpene β-amyrin with anti-inflammatory, hypolipidemic, hypoglycaemic, and many other physiological functions, currently extracted from 3- to 5-year-old liquorice *Glycyrrhiza glabra*, were produced from 1000 kg of *Magnolia officinalis* residues [27].

## 5. Honokiol in Experiments

The broad range of the beneficial pharmacological properties and phytotherapeutic effects of honokiol [63,64,65,66,67] have attracted scientific interest as a natural active constituent of cosmeceutical formulations [11,15,68,69,70]. The mechanisms of honokiol action and its therapeutic implications in various metabolic pathways have aroused growing interest as well [13,17,23,71,72]. Studies on honokiol extracted from various organs of *Magnolia* plants have been conducted in many biological models: cell lines [13,73,74], animal studies [10,31,75,76,77,78], and clinical studies [79,80,81]. A wide range of pharmacological activity of honokiol, e.g., in chronic dermatoses, and its use in skin care have been documented [2,10,12,14,21,73,74,75,76,77,78,79,80,81,82,83]. Currently, the search focuses on new natural, bioactive chemical compounds, such as honokiol, with the therapeutic potential to support conventional treatment [8,84,85] and ensure safety for humans and animals without side effects [26,79,85].

## 6. Honokiol Content in Selected Organs of Several *Magnolia* Species

Bioactive chemical compounds with health-enhancing effects are extracted from various organs of many *Magnolia* species. The group of lignans in these plants are represented by neolignans, e.g., honokiol and its structural isomers 4-O-methylhonokiol and obovatol, which have similar properties, and magnolol, 3-methoxymagnolol, isomagnolol, tripetalin, magnoquinone, and magnotriol [52,86,87]. Honokiol is extracted from various raw materials of many *Magnolia* species [4,5]. It has been identified in, e.g., *M. acuminata* [88], *M. champaca* [52,89], *M. dealbata* [5,24], *M. denudata* [52,90], *M. fargesii* [91], *M. grandiflora* [24,52], *M. hodgsonii* [92], *M. obovata* [24,93], *M. officinalis* [27,52,84], *M. officinalis* var. *biloba* [79,94], *M. pterocarpa* [95], *Magnolia×soulangeana* [96], *M. tripetala* [86,94], *M. virginiana* [97], and *M. kobus* [98,99].

The content of honokiol varies between the different organs of *Magnolia* plants. This effective therapeutic agent with a wide spectrum of biological activity has been detected in *Magnoliae gemmae* [5], *Magnoliae flos* [79,94], *Magnoliae semen* [90], *Magnoliae folium* [94], and *Magnoliae cortex* [84,94,100,101]. Its concentration in *Magnolia* species varies depending on type of the raw material (Table 1).

## 7. Structure and Physicochemical Properties of Honokiol

Honokiol (5,3′-Diallyl-2,4′-dihydroxybiphenyl; C_18_H_18_O_2_) has been applied for centuries in the therapy of inflammatory diseases, food indigestion, typhoid fever, depression, and insomnia [8,17,103,104,105,106]. This lignan biphenol is generated in the shikimic acid pathway [107]. It is an isomer of magnolol (bearing an isomeric bisphenol core substituted with allyl functions) with a molar mass of 266.33 g·mol^−1^, a melting point of 87.3 °C, an evaporation point of 400 °C, and a heat of evaporation of 67.7 kJ·mol^−1^ [7,108,109,110,111]. In terms of the physical form, it is a white, crystalline powder with high dissolvability in organic solvents and moderate solubility in water [11,16,90]. As a small-molecule lipophilic compound with broad biological activity, honokiol is suitable for topical use [2]. In chemical terms, this polyphenol from the lignan group represents biphenyl-type neolignans, in which two phenylpropene units, para-allylphenol and ortho-allylphenol, are linked via a C-C bond between aromatic rings [2,86] (Table 2).

The small size of honokiol particles and its good solubility in fats facilitate its penetration through cell membranes and the blood–brain barrier; hence, honokiol exhibits considerable bioavailability, e.g., in nervous tissues [17]. Additionally, honokiol is hydrophobic and interacts with proteins in cell membranes, crossing physiological barriers, which ensures its high bioavailability [117]. As a phytoestrogenic lignan with health-enhancing properties [65,118,119,120], honokiol exerts beneficial effects in the treatment of dermatological conditions [2,7,121].

## 8. Therapeutic Properties of Honokiol in Certain Diseases

**Figure 2:**
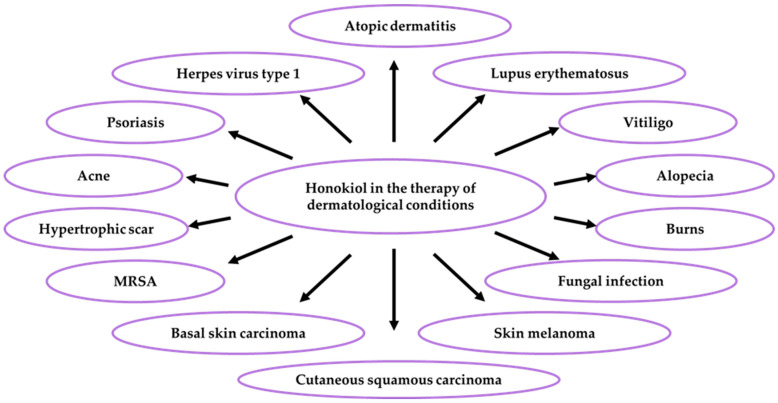
Use of honokiol in dermatological conditions [3,14,73,123,126,129,130,131,132,133,134,135,136,137]. Note: methicillin-resistant *Staphylococcus aureus* (MRSA).

In terms of phytotherapeutic properties, honokiol exhibits its health-enhancing properties in various diseases, e.g., dermatological [2,21] or immunological [122] conditions. The effectiveness of honokiol has been documented in the therapy of psoriasis [2,123,124], atopic dermatitis [3], and UV radiation-induced skin cancers [125,126]. The therapeutic effects of honokiol have been assessed in malignant melanoma [73,121] and keratinocyte cancers: basalioma [127] and epidermoid carcinoma [126]. Honokiol has also been used in the treatment of oral, as well as head and neck, squamous cell carcinoma [128,129]. No mutagenic or genotoxic effects were observed after honokiol application *in vitro* and *in vivo*, and the safety of its use was confirmed by food safety organisations [2]. The use of honokiol in various skin diseases is presented in Figure 2.

## 9. Selected Biological Properties of Honokiol

Honokiol exhibits a wide range of phytotherapeutic properties, e.g., antimicrobial [138,139,140], antiviral [135], antibacterial [132,133,138], and antifungal [81,139] effects, as well as neuroprotective [140,141], antioxidant [142], anti-inflammatory [68], antifibrotic [143,144], antidepressant [104,145], and anticancer activity [73,78,117].

Currently, research is being conducted in various models to potentially use the biological properties of honokiol in the phytotherapy of dermatological problems. Honokiol has been evidenced to exert a strong effect on some pathogens, including those from the families Herpesviridae and Papillomaviridae, causing skin diseases [8,137,146].

### 9.1. Antiviral and Virucidal Activity of Honokiol

The antiviral activity of honokiol against viruses causing skin diseases, including herpes simplex virus type 1 (HSV-1), has been documented [2,137]. Honokiol reduced HSV-1 DNA replication, gene expression, and production of new progeny viruses. In turn, honokiol applied in combination with acyclovir enhanced the inhibition of HSV-1 infection. This indicates that honokiol can be used alone or combined with other drugs in the therapy of HSV-1 infection [137]. Other studies demonstrated that, even at low concentrations, honokiol reduced the expression of HSV-1 viral protein, thereby counteracting infection with herpes simplex virus-1 (HSV-1), with an inhibition rate exceeding 90%, which confirms its potential to be used in the treatment of this disease [8].

**Figure 3:**
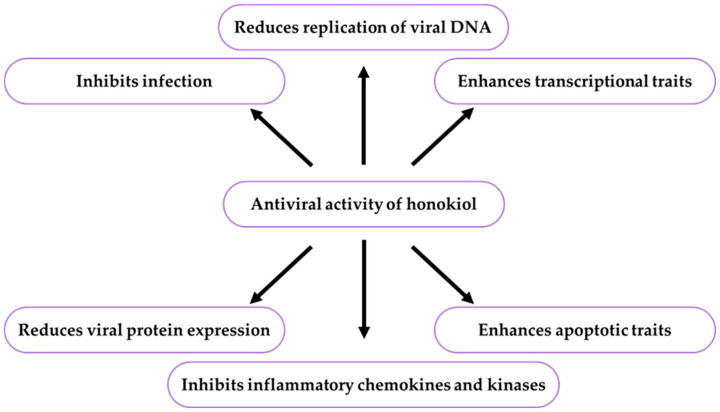
Mechanism of the antiviral action of honokiol against herpes simplex virus type 1 (HSV-1) and human papillomavirus (HPV).

Honokiol and other bioactive chemical compounds, such as amentoflavone, apigenin, baicalin, berberine, bergapten, formononetin, genistein, glycyrrhizic acid, hesperidin, kaempferol, luteolin, rutin, scutellarin, spathulenol, and quercetin, can be used alone or as components of drugs against human papillomavirus (HPV) infections [147]. Honokiol liposomes were found to be effective in treating flat warts caused by HPV infection. After honokiol was topically applied, its therapeutic effect, with only minor side effects, was observed [8]. In the therapy of COVID-19, honokiol exerted an impact on apoptotic and transcriptional features, levels of chemokines and kinases, and amounts of adhesion molecules on cell surfaces [17]. The antiviral effects of honokiol in selected biological models are shown in Table 3 and Figure 3.

In addition to its antiviral properties, honokiol may be an effective phytocompound with antibacterial activity to be used in the treatment of various skin diseases caused by Gram-positive and Gram-negative bacteria [20]. Mainly allyl groups and hydroxyl groups on the benzene ring are the important determinants of the antibacterial activity of honokiol and its derivatives against bacterial pathogens that cause skin diseases [8]. Currently, skin infections caused by drug-resistant bacteria pose a serious public health problem and are part of a new research trend in anti-infectious disease treatment. In this therapy, honokiol and honokiol amphiphiles may be effective components of new antibacterial drugs, potentially reducing bacterial skin infections through biofilm-destroying mechanisms [153,154]

### 9.2. Antibacterial Activity of Honokiol

**Figure 4:**
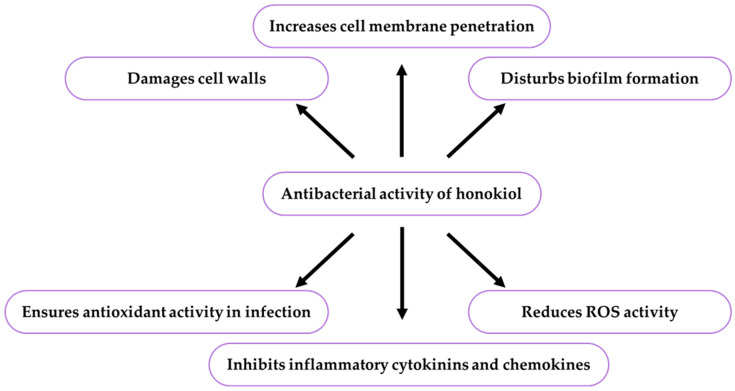
Mechanism of the antibacterial action of honokiol against bacterial strains causing dermatological diseases: *Porphyromonas gingivalis*, *Prevotella intermedia*, *Propionibacterium acnes*, *Pseudomonas aeruginosa*, and *Staphylococcus aureus.* Notes: ROS—reactive oxygen species.

Honokiol exerted a pleiotropic effect on certain metabolic pathways and organism functions and reduced inflammation lesions and free radicals [155]. Extracts of four *Magnolia* species, *M. grandiflora*, *M. champaca*, *M. officinalis,* and *M. denudate,* effectively inhibited the Gram-negative bacterial strains *Prevotella intermedia*, *Porphyromonas gingivalis*, and *Pseudomonas aeruginosa* and the Gram-positive bacteria *Propionibacterium acnes* and *Streptococcus faecalis* [52]. The resistance of *Staphylococcus aureus* to methicillin indicates the necessity to search for new immunomodulatory phytocompounds preventing infections by these bacteria. Literature data confirm that honokiol and magnolol have effective health-enhancing cell immunomodulatory activity during *S. aureus* infection [156]. Currently, the interest in natural care cosmetics is increasing, hence the great demand for phytochemicals that can be used as natural preservatives instead of parabens, which are known to cause side effects. The ethyl 1-acetoxyethane fraction from *M. obovata* and *Lonicera japonica* exerted a potent antibacterial effect against strains of monoderm bacteria *Staphylococcus aureus* and *Bacillus subtilis* and diderm bacteria *Pseudomonas aeruginosa* and *Escherichia coli* and had fungistatic and fungicidal activity against *Candida albicans* and *Aspergillus brasiliensis* strains. This activity was related to magnolol and honokiol interactions in *M. obovata* and caffeic acid and luteolin in *Lonicera japonica*. In the combined application of extracts from *M. obovata* and *L. japonica*, these compounds can be a natural preservative of plant origin [157]. The antibacterial effects of honokiol on some bacterial strains are presented in Table 4 and Figure 4.

#### Effect of Honokiol on Biofilm Formation

Honokiol has been shown to be effective against biofilm formation [153]. The extracellular material produced by microorganisms, called the matrix, constitutes over 90% of the biofilm and consists of extracellular polymeric substances, including polysaccharides, proteins, extracellular DNA, and lipids, which prevent antimicrobial agents from penetration. Honokiol exhibits effective antibacterial activity through inhibition of biofilm formation by cariogenic oral pathogens, e.g., *Enterococcus faecalis*, *Porphyromonas gingivalis*, *S. aureus*, and *S. mutans*. Low toxicity is desirable for antimicrobial agents to be used in oral health care. Some compounds contained in magnolia bark are characterised by low genotoxicity and anticlastogenic activity (*in vivo*) [160], as well as low toxicity towards human epithelial cells and fibroblasts [153,160]. Another group of honokiol-susceptible pathogenic microorganisms comprises the acne-causing bacteria *Propionibacterium acnes* and *P. granulosum*, which trigger inflammation of sebaceous glands or hair follicles. Honokiol and magnolol exhibit strong antibacterial and anti-inflammatory activity against these bacteria without causing skin irritation. This indicates that they can be used as effective therapeutic agents for the alleviation of acne symptoms [153]. The phytotherapeutic effects of honokiol also include the elimination of biofilm produced by *S. aureus*. Isolates of this strain are capable of formating various types of biofilm mediated by polysaccharide intercellular adhesion (PIA) or extracellular DNA (eDNA). Honokiol can detach formed biofilms, kill bacteria present within biofilms, and simultaneously inhibit the sarA, cidA, and icaA transcript levels, as well as eDNA release and PIA expression [162]. It effectively destroys *Acinetobacter baumanii* and *Vibrio cholera* via inhibition or removal of existing biofilms and limitation of the motility of these antibiotic-resistant bacteria [159].

In addition to the biological activities of honokiol described so far, this phytocompound exerts antidermatophytic effects against multidrug-resistant fungal pathogens that cause skin diseases in humans. Dermatophyte skin infections have been currently shown to be a serious problem. Among many bioactive compounds, honokiol is a promising drug ingredient in therapies of dermatological conditions, as it has been comprehensively investigated in various biological models [2,7,164]. Information about the effect of honokiol on biofilm formation is shown in Table 4.

### 9.3. Antifungal Activity of Honokiol

Honokiol had a beneficial effect on metabolic processes by reducing the symptoms of keratomycosis caused by *Aspergillus fumigatus*, had antifungal and anti-inflammatory activity, and inhibited fungal keratitis (*ex vivo* and *in vivo*). The MIC_90_ and the minimum fungicidal concentration (MFC) values of honokiol were 8 and 12 µg·mL^−1^, respectively. Honokiol exerted its fungistatic effect after 6 h and a fungicidal effect after 24 h without any cytotoxicity. It inhibited *A. fumigatus* growth, adhesion, and biofilm formation by enhancing the pathogen cell membrane permeability. In C57BL/6 mice, it reduced the disease severity, neutrophil recruitment (MPO, IFS, and FCM), and fungal burden. It suppressed the expression of Toll-like receptor 2 (TLR-2) mRNA and protein, high mobility group box 1 (HMGB1) non-histone protein, and pro-inflammatory IL-1β and TNF-α. Honokiol may also serve as an anti-inflammatory component by promoting M2 phenotype polarisation and the amelioration of inflammation lesions through Dectin-2 downregulation. It may also be a promising therapeutic compound employed the for therapy of fungal keratitis [164,165].

**Figure 5:**
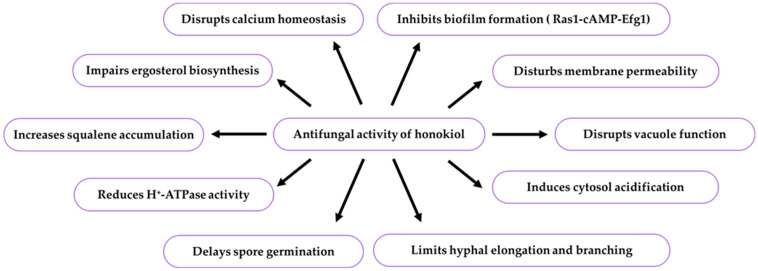
Mechanism of the antifungal action of honokiol against fungal strains causing dermatological diseases: *Aspergillus* spp., *Candida* spp., *Microsporum* spp., and *Trichophyton* spp. Notes: signalling pathway (Ras1-cAMP-Efg1).

*Candida* yeasts cause acute or subacute fungal infection, i.e., cutaneous candidiasis [166]. There are more than twenty species of *Candida* yeasts, including *Candida albicans*, which can trigger mucosa and skin infections in immunocompromised patients [167]. Honokiol inhibited *C. albicans* growth, reduced the level of ergosterol (ergosta-5,7,22-trien-3-ol), and increased the expression of genes involved in the ergosterol biosynthetic pathway. It caused abnormalities in vacuole morphology and function, disturbed intracellular calcium homeostasis, inhibited ergosterol biosynthesis, and reduced Pma1p H^+^-ATPase (proton pump) activity. This resulted in untypical pH in the vacuole and cytoplasmic matrix of the pathogen. The antifungal effects were related to the activation of the calcineurin (protein phosphatase 2B) signalling pathway, engaged in honokiol tolerance [168]. Additionally, honokiol can inhibit biofilm formation by the mitosporic fungus *C. albicans* through reduction of adhesion, morphological transformation, and fungicidal activity. This confirms the potential of this compound to be used as a part of the therapy of infections caused by this microorganism [59]. The antifungal activity of honokiol is presented in Table 5 and Figure 5.

The antidermatophyte activity of honokiol is associated with its strong antioxidant and anti-inflammatory properties. It inhibits, e.g., IL-1β, IL-8, and TNF-α and slows down the mitogen-activated protein kinase-1 (MEKK-1) pathway in signalling the activation of factor kappa light chain of activated B cells (NF-κB), which is crucial for its anti-skin disease effects (anti-psoriasis activity). It also has anticancer properties, as MEKK-1 is a component of the stress-activated protein kinase/c-Jun N-terminal kinase pathway. It is a key constituent of the RAS/RAF/MEK/MAPK signalling pathway, which regulates cell proliferation and is frequently activated in human cancers [7,68].

### 9.4. Anti-Inflammatory Activity of Honokiol

One of the causes of skin damage is air pollution, including particles of soot, smoke, and acids, which disrupt the structural integrity of the skin, interact with the skin microflora, and cause or exacerbate skin diseases [169]. Honokiol plays an important role as one of the wide range of phytochemicals used in dermatological therapy [2]. Honokiol applied at doses of 10 and 20 µM to human HaCaT keratinocyte lines exposed to cigarette smoke reduced IL-8 levels by 24 and 53% and apoptosis by 47 and 41%, respectively. In HFF-1 fibroblasts, it restored the production of the tissue inhibitor of metalloproteinases 2 (TIMP-2) (97 and 92%) and reduced the expression of β-galactosidase. Additionally, honokiol inhibited IL-8 and IL-1α synthesis in keratinocytes. It also prevented apoptosis and had anti-collagenolytic, anti-aging, and anti-inflammatory effects [9].

Transient receptor potential vanilloid-superfamily member 3 (TRPV3) channels are involved in various physiological processes: pruritus and temperature sensing, wound recovery, epidermal barrier maintenance, embryogenesis, and anagen promotion. They are also implicated in dermatoses, e.g., Olmsted syndrome (periorificial keratoderma), atopic dermatitis (eczema), and acne rosacea. Investigations of TRPV3 are focused on identification of active compounds targeted at these channels [170]. Honokiol and magnolol inhibit TRPV3 with simultaneous reduction of IL-6 and IL-8 release. They also inhibit overactive TRPV3 variants in epidermal keratinocytes, and these properties are useful in therapies for inflammatory skin diseases [21].

**Figure 6:**
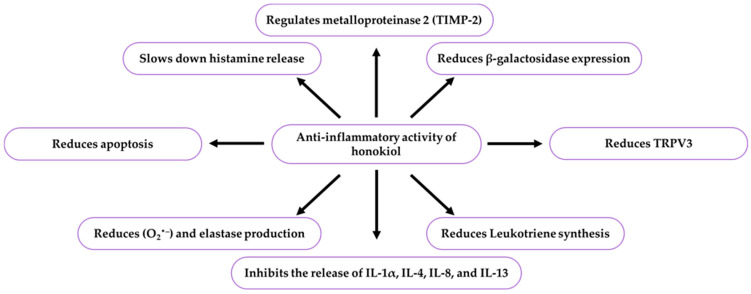
Mechanism of the anti-inflammatory action of honokiol. Notes: superoxide anion radical (O_2_^•−^), tissue inhibitor of metalloproteinases 2 (TIMP-2), interleukin (IL).

A honokiol dose of 10 mg·mL^−1^ limited abnormal epidermal proliferation and accumulation of mastocytes, also known as Ehrlich cells or labrocytes, and inhibited the level of excessive inflammatory cytokines (IL-4, IL-3, chemokines CCL17 and CCL22) in the epidermis and lymph nodes in mice. It alleviated inflammation and inhibited histamine release and leukotriene synthesis in mast cells. It also exerted antioxidant and anti-inflammatory effects, and these properties can be useful for the supportive therapy of atopic dermatitis [3]. In other studies, honokiol and its derivative (2-O-acetyl-4′-O-methylhonokiol) limited the priming and activation of neutrophils and macrophages through suppression of superoxide radical anions (O_2_^−^), a serine protease elastase also known as polymorphonuclear elastase or PMN elastase, and interferon-*β-2*, also named IL-6, hybridoma/plasmacytoma growth factor, B-cell stimulatory factor 2, CTL differentiation factors, or hepatocyte stimulating factor. Compared to the derivative, honokiol was characterised by higher activity and lower cutaneous absorption. These compounds can be used as safe ingredients of formulations for topical treatment of skin inflammatory conditions [12]. The antiphlogistic effects of honokiol in some biological models are presented in Table 6, Table 7 and Table 8 and Figure 6.

Nanotechnology is used for development of modern pharmaceutical and cosmetic products, with the use of carriers increasing the effectiveness of the bioactive compound and the product. The available honokiol nanocarriers are characterised by greater water solubility and stability, greater efficacy, higher bioavailability, and more potent *in vitro* and *in vivo* anti-inflammatory effects on the skin than the pure compound. This indicates that nanosuspensions can be used in anti-inflammatory phytotherapy of the skin [2,68]. As reported by Li et al. [2], since honokiol is a small, lipid-soluble molecule, it is suitable for topical use. It has a broad spectrum of action, and its topical application is a safe and effective therapeutic method in dermatology.

Targeting inflammatory mediators is one of the key elements playing a fundamental role in the honokiol-induced inhibition of skin photoaging, appearance of pigmentation changes, and discoloration. It is also crucial in the photoprotection process and prevention of UVB-induced skin cancer (basalioma, squamous cell carcinoma, and malignant melanoma) [2,9,10].

### 9.5. Photoprotective Activity of Honokiol

Ultraviolet radiation, mainly the ultraviolet-B radiation (UVB) spectrum, is a carcinogenic factor in skin cancers [176]. Skin exposure to ultraviolet radiation (UVR) was reported to inhibit allergic reactions in contact hypersensitivity (CHS) and impair the immune system, which may pose a risk of skin cancer [177]. Effective phytochemicals that will delay UVB-caused suppression of immune sensitivity and exhibit effectiveness in anticancer therapy are still being sought. Topical application of honokiol to UV-irradiated mouse skin inhibited inflammatory mediators and stimulated immune reactivity via DNA demethylation-dependent functional activation of accessory cells [176,177].

**Figure 7:**
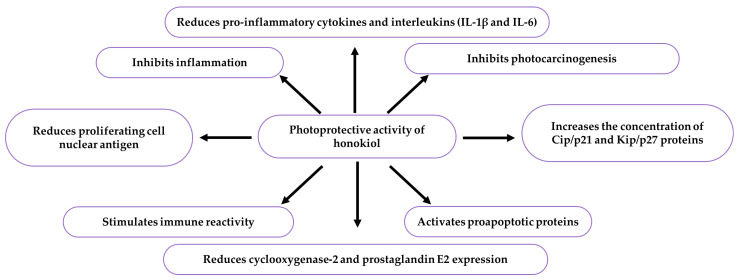
Mechanism of the photoprotective action of honokiol. Notes: interleukin (IL).

Honokiol applied topically as a hydrophilic cream onto the skin of tumour-bearing SKH1-Hrhr mice before or after UVB irradiation inhibited photocarcinogenesis (28–60%) and tumour volume (33–80%). It reduced the expression of prostaglandin–endoperoxide synthase 2 (cyclooxygenase-2, COX-2), prostaglandin E2 (dinoprostone), proinflammatory cytokines and interleukins (IL-1β and IL-6), tumour necrosis factor, and proliferating cell nuclear antigen. The levels of cyclins (D1, D2, E), kinases (CDK2, 4, and 6), phosphatidylinositol 3-kinase, and Akt Ser 473 phosphorylation were reduced, whereas the levels of apoptosis-regulating proteins Cip/p21 and Kip/p27 were higher. Honokiol slowed down inflammatory mediators, cell cycle progression, and survival factors, which indicates that it can be used in the therapy of UV-mediated skin cancers [178]. Honokiol had a chemopreventive effect on the development of UVB-caused skin cancer in SKH1-Hrhr mice. It reduced the proliferation of tumour cells (45%). The concentration of caspase-3, -8, and -9 and poly(ADP-ribose) transferase, also known as poly(ADP-ribose) polymerase (PARP) and the activation of p53 led to the induction of DNA fragmentation and programmed cell death. The application of honokiol (30 µg) prevented further progression of skin cancer through activation of proapoptotic proteins [179]. Topically applied honokiol (30, 45, and 60 µg) prevented UVB-induced skin cancer in SKH1-Hrhr mice. It is therefore a safe and powerful therapeutic agent [156]. Prevention of skin exposure to UV radiation, including a frequent use of sun creams, reduces the incidence of such cancers as skin melanomas [180]. The photoprotective effects of honokiol are presented in Table 9, Figure 7.

Targeting inflammatory mediators is one of the key elements playing a fundamental role in the honokiol-induced delay in the skin photoaging process or appearance of pigmentation changes and discolorations and in photoprotection and prevention of UVB-induced skin cancer (basalioma, squamous cell carcinoma, and malignant melanoma) [2,9,10].

### 9.6. Anticancer Activity of Honokiol

Honokiol anticancer effects through various metabolic pathways [129] have been documented in the treatment of various cancers types: leukaemia [182,183], lymphoma [184,185], glioblastoma multiforme [186,187], ovary cancer [188,189], oral cavity cancer [145,190], colon cancer [191], nasopharynx cancer [119,192], kidney tumours [193], bladder cancer [31,194], lung cancer [195,196], breast cancer [90,197], prostate cancer [198,199], skin cancer [73,200], thyroid cancer [201,202], pancreas cancer [78,203], hepatocellular carcinoma [192], other liver tumours [144,204], osteosarcoma [205,206], multiple myeloma [109], oral cancer [128], and head and neck epidermoid carcinoma [196,207,208].

### 9.7. Activity in Skin Cancer Treatment

The therapy of skin cancer, one of the most prevalent neoplasms, involves the use of active chemical compounds, e.g., such phytochemicals as honokiol, to prevent cancer initiation or progression [209,210,211]. Using various experimental models, the anticancer properties of honokiol were studied in two main subgroups of skin malignancies: melanoma [73,74] and non-pigmented skin cancers, including rodent ulcer, commonly known as basalioma [126], and squamous cell (epidermoid) carcinoma [129,212], as well as rare, aggressive neuroendocrine carcinoma, also named Merkel cell carcinoma [213]. The honokiol anticancer properties confirmed in numerous studies indicate its potential use as a future chemotherapeutic agent [210,211,214]. The anti-oncogenic efficacy of honokiol is associated with the regulation of epithelial and mesenchymal markers, the modification of some oncogenesis-related signalling pathways, and cell cycle arrest via cyclic protein regulation. Honokiol was found to prevent metastasis by limiting cell migration and invasion through regulation of matrix metalloproteinases [117].

#### 9.7.1. Activity Against Melanoma

Skin melanoma is one of the most malignant tumours originating from neuroectodermal melanocytic cells [215,216]. In 2020–2022, melanoma accounted for 5% of all diagnosed malignant tumours in Europe and 2% in Poland, where it is the cause of 1.4% of cancer-related deaths [217]. Studies on skin melanoma are focused on the search for therapeutic chemopreventive agents with effectiveness towards cancer cells and without side effects on the organism. In addition to the commonly used treatment options, many experiments aim at finding effective natural bioactive chemical compounds for chemoprevention of malignant melanoma [218]. Melanomas are cancers with limited therapeutic options [74]. The treatment is based on the use of a BRAF inhibitor (vemurafenib), but the disease often tends to relapse. Therefore, new therapeutic approaches characterised by low toxicity are being sought. One of them is honokiol. With its activity against solid tumours and haematological malignancies [219], honokiol is regarded as an effective anticancer agent [73,74,121,220,221]. It exerted cytotoxic and cytostatic effects in malignant melanoma cells [2,13,85]. Since honokiol is characterised by very low oral bioavailability, topical application is an effective mode of administration thereof [74]. It slowed down the invasion of melanoma cells via lowering the concentration of the oncogenic protein 18 (KRT18) and degradation through ubiquitination, which consequently limited *in vitro* and *in vivo* growth of melanoma cells [73]. Honokiol limited the invasiveness of melanoma cell lines Hs294t and SK-Mel 28 and suppressed nicotinamide adenine dinucleotide phosphate (NADPH 1) oxidase activity. It reduced the binding of p22phox core proteins to p47phox proteins via increased accumulation of cytoplasmic p47phox protein and reduced expression of membrane-bound p22phox protein (*in vitro*). It acted as an inhibitor of NADPH oxidase 1 and melanoma cell migration. Honokiol may be a component of chemopreventive or therapeutic strategies against melanoma [222]. NADPH oxidase 1 (Nox1) overexpression in melanoma cells is often connected with an elevated metastasis rate. Honokiol inhibited the migration of human melanoma cell lines (SK-Mel119, SK-Mel28, Hs294t, and A375) and reduced oxidative stress and NADPH oxidase activity by blocking the interactions between cytosolic and membrane-bound proteins involved in ROS generation [126].

Honokiol inhibited stem cell division, melanoma cell proliferation, viability, and clonogenicity and induced autophagy. It reduced the formation of melanospheres and the expression of Notch-2 receptors and target proteins, Hes-1 and cyclin D1, and the expression of TACE protein complex and proteolytic enzymes (secretase) in melanoma cells. Honokiol is a potent inhibitor of melanoma cells through inhibition of Notch-2 signalling [220]. The increase in the incidence of skin melanoma suggests the necessity of devising new therapeutic strategies, e.g., involving the WNT/β-catenin signalling pathway and the MITF transcription factor responsible for melanoma cell invasion. Honokiol inhibited melanoma growth and metastasis, induced CHOP activation in ER stress, and reduced the expression of MITF, β-catenin, and CDK2 in melanoma tissues (*in vitro, in vivo*). It exerted an impact on the activation of CHOP/GADD153, regulating the activation of β-catenin, increased the interaction of calpain-10 and MITF-m, and caused MITF-m cleavage. The high expression of MITF, β-catenin, and CDK2 indicates that honokiol is an efficient melanoma inhibitor [121]. In murine malignant melanoma models, honokiol reduced cell viability, proliferation, and cell cycle arrest. It increased apoptosis and modulation of apoptosis and cell cycle regulatory proteins (*in vitro*). Honokiol contributed to the accumulation of cells in the G2/M phase of the cell cycle (SKMEL-2 cells) and in the G0/G1 phase (UACC-62 cells). Both honokiol-treated melanoma SKMEL-2 and UACC cells exhibited increased levels of caspases and PARP, reduced expression of cell cycle regulatory proteins, and weakened tumour growth and progression in SKMEL-2 and UACC-62 melanoma xenografts [221].

Two synthesised honokiol compounds (honokiol dichloroacetate ester (DCA) and bis-trifluoromethyl-bis-(4-hydroxy-3-allylphenyl)methane (hexafluoro)—a fluorinated analogue of honokiol) with increased lipophilicity were used against melanoma A375 (*in vivo*) and increased the induction of the succinate dehydrogenase B (SDHB) respiratory enzyme. Honokiol DCA was more active against LM36R melanoma (vemurafenib-resistant). Both compounds inhibited DRP1 phosphorylation and normalised mitochondrial function. Additionally, honokiol DCA intensified respiration and reactive oxygen generation in vemurafenib-resistant melanomas with an effect against aggressive melanoma *in vivo* [219].

**Figure 8:**
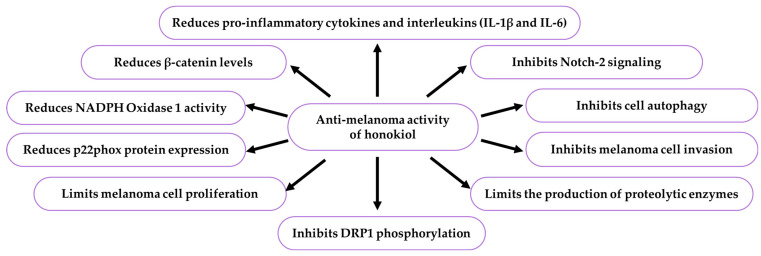
Mechanism of the anti-melanoma action of honokiol. Notes: nicotinamide adenine dinucleotide phosphate (NADPH), dynamin-related protein 1 (DRP1).

The carrier system consisting of honokiol transfersomes (190 nm) with high negative charge and drug entrapment efficiency (EE%) used in the skin-directed therapy of melanoma prolonged the honokiol release time (48 h) and overcame horny layer (stratum corneum) physiological barriers and the tumour microenvironment. Honokiol transfersomes also alleviated the immunosuppressive properties of B16F10 melanoma through regulation of TGF-β signalling and reduction of the expression of the CD47 signal (*in vitro*). They limited the expression of the stem cell marker CD133 (Promitin-1) and the level of CD47, CD133, and TGF-β ameliorating immunosuppressant properties of melanoma stem cells. An increase in curative effects was noted upon metformin but not collagenase pretreatment [74]. The properties of honokiol used in melanoma therapy in some biological models are presented in Table 10, Figure 8.

#### 9.7.2. Activity Against Non-Melanoma Skin Cancers

Keratinocyte cancers differ in their appearance and exhibit a varied pigmentation degree [204,205,206,207]. They most often affect fair-skinned subjects [208,209]. The group of non-pigmented skin cancers comprises basalioma [204], originating from non-keratinising cells of the epidermis stratum basale [210], squamous cell carcinoma [208,211,214], originating from cells of the epidermis stratum spinosum [212], and neuroendocrine Merkel cell carcinoma [196,213].

##### Honokiol in the Treatment of Basalioma

Honokiol inhibits tumour growth and promotes apoptosis in cancers. The authors of [140] investigated the effect of honokiol and its combined use with the anticancer 5-fluorouracil (5-FU) medication on skin cancer in two cell lines: basal cell carcinoma cells (TE 354.T) and immortalised human keratinocytes (HaCaT). Honokiol doses of 10 µg·mL^−1^ effectively suppressed cell growth and division and induced cell apoptosis, while the opposite effect on HaCaT keratinocytes was shown when this polyphenolic neolignan was combined with 5-FU.

##### Honokiol in the Treatment of Squamous Cell Carcinoma

Chemotherapy resistance is a problem in cancer therapy. Systemic chemotherapy is hampered by rapid drug removal from the circulation, a low drug concentration at the tumour site, and undesirable side effects [215,216]. Honokiol used in cancer therapy limited tumour growth through apoptosis and inhibition of angiogenesis [150,217]. It reduced the transformation of papillomas into tumours without *in vitro* and *in vivo* side effects. Honokiol strongly affected inflammatory mediators and signalling pathways, i.e., cell survival signalling, nuclear factor-κB (NF-κB), signal transducer and activator of transcription 3 (STAT3), epidermal growth stimulus receptor (EGFR), cell division, and prostaglandin-endoperoxide synthase (cyclooxygenase). It also exerted chemopreventive effects in the treatment of malignant melanoma, keratinocyte cancer, and head and neck epidermoid carcinoma [139]. A study of the chemopreventive efficacy of honokiol on buccal pouch squamous cell carcinoma in hamsters showed changes in the area fraction of collagen fibres, inhibition of invasion, proliferative activity, and tumour advancement [194].

Honokiol limited the development of human oral squamous cell carcinoma (OECM-1 or OSCC) cell lines. It decreased cell proliferation, slowed down the cell cycle at phase G1 and the Cdk2 and Cdk4 concentration, and increased cell cycle inhibitors p21 and p27. It caused caspase-dependent apoptosis and autophagy regulated by an autophagic inducer, rapamycin or bafilomycin, which increased the anti-cancer effect. It inhibited the MAPK pathway and the regulation of Akt/mTOR or AMPK pathways. Honokiol synergistically improved the therapeutic cell-killing effect of 5-FU. Upon oral administration, it exerted antitumour effects in OSCC xenograft mice. Honokiol inhibited OSCC growth by inducing programmed cell death, cell cycle arrest, and autophagocytosis (*in vitro* and *in vivo*) [142]. Honokiol applied to human head and neck (HNSCC), buccal cavity (SCC-1), larynx (SCC-5), tongue (OSC-19), and pharynx (FaDu) cancer cell lines inhibited cell viability through induction of apoptosis, correction of dysregulated G0/G1 phase cell cycle proteins, and reduction of the expression of epidermal growth factor receptor (EGFR) and mTOR. Honokiol (100 mg·kg^−1^ body weight) limited SCC-1 and FaDu xenograft growth in mice and rapid multiplication of tumour cells, enhanced induction of programmed cell death, and inhibited the EGFR signalling pathway and the expression of cyclins and Cdk. The therapeutic effect of honokiol against HNSCC resulted from the stronger binding of EGFR than gefitinib, a drug used in HNSCC treatment [143]. Honokiol applied to OSCC cell lines HN22 and HSC4 decreased the expression of the enzyme nitric oxide synthase (iNOS) and the level of the ERp44 ER-resident protein and inhibited the proliferation of tumour cells and colony formation. Additionally, it limited inflammation and tumour growth in BALB/c mice with HN22 cell xenografts and initiated apoptosis through suppressing the *in vitro* and *in vivo* expression of iNOS/NO and ERp44 in the HSC4 and HN22cells and xenograft tumours. Honokiol may be a component of anti-inflammatory and anticancer drugs in the therapy of OSCC in humans [141].

## 10. Future Directions

Honokiol PI3K/Akt signalling inhibitors, the main targets of anticancer therapy, involved in the development of tumours, should be investigated in future research experiments on various cell lines. This issue is currently arousing scientists’ interest and is the current topic of research. Further clinical studies are necessary to elucidate the signalling pathways and molecular mechanisms of the inhibitory effect of honokiol on, e.g., cancer. The purity, standardisation, toxicity, and safety level of honokiol in biological and synergistic reactions with other bioactive chemical compounds should be investigated. It is also necessary to clarify the mechanisms of its synergistic effects at the cellular and molecular levels, which will help researchers to design a safe and successful therapy. Furthermore, further studies of honokiol as a non-toxic angiogenesis inhibitor are recommended, with the aim of using this compound as a potential chemotherapeutic agent. Honokiol studies are still limited to experiments in various *in vitro* models. Currently, there are only a few reports confirming the safety and efficacy of honokiol used as a drug in human medicine. Therefore, further investigations with clinical studies are necessary to verify the targeted health-enhancing role of honokiol in specific pharmacological outcomes, taking into account changes in the function of the cell, organ, and whole organism and searching for modes of administration of appropriate doses of the compound. The search for the possibilities of using honokiol in the therapy of some cancers should be expanded to include mechanistic pathways of cancer inhibition. A new trend for future research is the development of methods for delivering honokiol by nanocarriers at the cellular level and validation thereof in various biological models and clinical studies. Highly important in this regard is the strategy of the delivery of honokiol nanoformulations via exosomes obtained from other cells, which is a prospective trend for development of honokiol-delivery nanosystems and enhancement of synergistic effects and therapeutic selectivity in some diseases. These tasks require more comprehensive experimental and clinical studies to be carried out in future research projects.

## 11. Materials and Methods

### 11.1. Review Design

This review presents the outcomes of exploration of literature data on the properties of honokiol contained in various *Magnolia* species used in the phytotherapy of many human and animal diseases, with particular emphasis on selected dermatological problems. It describes bioactive chemical compounds contained in various raw materials from *Magnolia* plants as well as the chemical and physical properties and mechanisms of action of honokiol applied in certain dermatological conditions and investigated in various biological models. Information on the bioavailability and biological and therapeutic effects of honokiol used as a phytotherapeutic ingredient is presented in this review as well. It is based on searching in specialised research databases of contemporary studies of the biological properties of honokiol in various biological models and its use in the therapy of dermatological problems. The following issues are discussed in this review: (1) the content of honokiol in various raw materials of various *Magnolia* species, (2) the structure and physical, chemical, and biological properties of honokiol, (3) its antiviral, (4) antibacterial, (5) antifungal, (6) anti-inflammatory, and (7) photoprotective effects, its activity against (8) melanoma and keratinocyte (basal and squamous cell carcinoma) cancers, and (9) its use in the therapy of some diseases.

### 11.2. Bibliographic Databases and Search Phrases

The review of the properties of honokiol and its use in phytotherapy of dermatological problems was conducted by searching 16 multidisciplinary specialised digital scientific and research databases: EBSCO (accessed date 14 April 2025), Nature Portfolio (accessed date 14 April 2025), ISI Web of Science (accessed date 14 April 2025), Google Scholar (accessed date 14 May 2025), Medline (accessed date 14 April 2025), Springer (accessed date 14 April 2025), Wiley Online Library (accessed date 15 April 2025), ProQuest SciTech Collection (accessed date 15 April 2025), ProQuest Central (accessed date 15 April 2025), PubMed (accessed date 15 April 2025), ScienceDirect (ac-cessed date 15 April 2025), Web of Science (accessed date 15 April 2025), Scopus (accessed date 16 April 2025), Taylor & Francis (accessed date 16 April 2025), and Web of Knowledge (accessed date 16 April 2025). The publications represented mainly the following research fields: biological, chemical, medical, agricultural, sociological, and social science. Original research publications strictly linked to the topic of the review were searched based on the following keywords: biological activity, apoptosis, chemical properties, physical properties, dermatological diseases, melanoma, dermatoses, antibacterial activity, antifungal activity, antimicrobial activity, phytotherapy, phytochemicals, photoprotective effects, honokiol, keratin, *Magnolia*, mechanisms of action, neuroprotective activity, neoplasms, honokiol derivatives, anticancer, antiviral, epidermoid carcinoma, basalioma, skin cancer, skin inflammation, oxidative stress, metabolic pathways, inflammation, and biologically active compounds. Our priority was to include the latest original high-quality articles based on their relevance to the discussed topics. Duplicates of identified publications available in different databases were eliminated.

### 11.3. Number and Dates of Publication of Articles

In total, 237 thematically consistent scientific reports are cited in this paper, including 230 original research papers, 6 chapters from various books, one doctoral thesis, and one literature reference concerning the chemical base. The literature sources for the main topic of the manuscript cover a period of 10 years (2015–2025). The review also includes five scientific publications providing essential data on the *Magnolia* taxonomy and the occurrence of plants of this genus in Poland; these articles were published in 2003, 2009, 2011, 2012, and 2013. Additionally, four publications from 2009, 2010, and 2012 are cited, as they explore the issue of UVB-induced skin cancers, including melanoma.

There is one publication on biofilm (2024) and six publications on honokiol extraction methods (2004, 2005, 2006, 2007, 2012). The group of literature from the last ten years comprises 219 articles, with 40% in the total number of reports published over the last five years and in this year. The analysis of the up-to-date knowledge of the properties and use of honokiol in dermatological problems is presented in descriptive, tabular (Table 1, Table 2, Table 3, Table 4, Table 5, Table 6, Table 7, Table 8, Table 9, Table 10 and Table 11), and graphic (Figure 1, Figure 2, Figure 3, Figure 4, Figure 5, Figure 6, Figure 7 and Figure 8, graphic abstract) forms. The tables present the results of studies on the concentration of honokiol in various *Magnolia* species, its physical and chemical properties, its antiviral, antibacterial, antifungal, and anticancer effects on the skin, the therapeutic doses used in various biological models, the duration of the experiments, and the research teams. In turn, the honokiol application in the therapy of fourteen dermatological disorders is presented in a diagram.

### 11.4. Citations of Literature References

The analysis of citations of the articles presented in this review was based on the Google Scholar internet search engine. The citations of the publications were sorted according to the consecutive years. All citation reads were downloaded on 26 June 2025. The citations of the publications analysed in this review are presented in Table 11.

**Table 11:** Number of citations of scientific publications as on 22.08.2025.

Year of Publication	Numerical Position of Publications Cited in the Manuscript	Citations Number of the Individual Publications	Total Citations Number in Each Year
2002–2005	[54,55] *,[59] *, [78] ^E^, [177] ***, [77] ^E^	17,16,13,133,332,9	520
2006, 2007, 2009,2010	[76,80] ^E^, [79] ^E^, [60] *, [145] **, [166,197] **	71,22,136,1,6,86,73,	392
2011–2013	[56] *, [61] *, [73] ^E^, [193] **, [62] *	0,1,0,13,0	14
2015	[108,114,123,128,148,156,158,180,207,220,222] ***	125,6,48,48,79,31,47,740,53,84,0,	1161
2016	[1,71,75,102,113,118,126,160,162,177,184,186,190,197, 198,219,223] ***	898,16,71,21,49,71,46,83,23,0,8,61,76, 32,29,36,36	1545
2017	[9,10,72,100,124,136,176,192,193,195,201,221] ***	29,45,95,187,0,37,0,59,44,2,63,24	585
2018	[11,12,15,79,129,157,167,172,173,174,199,202,218] ***	10,12,20,236,81,30,28,45,25,93,57,13,31	681
2019	[4,6,13,29,30,76,88,90,95,101,103,104,109,121,131,133, 137,145,159,187,188,189,194,205] ***	67,45,5,64,3,70,58,43,8,136,10,115,180, 44,7,49,29,10,36,77,48,3,24,0	1093
2020	[31,47,53,68,73,80,82,93,94,116,122,143,164,168,185, 224,225,226] ***	119,27,173,11,11,11,1,2,17,137,21,39, 39,19,6,5,21,1704	3023
2021	[7,16,20,23,24,40,46,48,69,74,86,107,110,115,132,140, 144,146,150,154,155,161,163,166,170,182,191,200,203, 204,227,228,229] ***	16,45,1,6,167,64,19,26,7,28,8,85,4,38,37,16,25, 20,16,100,47,50,6,21,12,39,28,0,30,20,316,12,18	2001
2022	[2,3,14,35,45,63,64,87,97,105,106,125,147,149,151,183, 206,212,213,217,230] ***	15,12,0,26,26,5,63,14,2,25,0,52,0,6,26, 3,23,4,0,10,0	312
2023	[5,17,18,26,32,54,56,65,77,81,84,89,96,98,111,117,119, 130,138,141,152,165,169,171,209,215,231,232,233] ***	4,35,16,19,5,5,20,9,15,9,8,34,4,3,6,15,32,23,7, 17,12,11,53,18,4,4,2,88,13	883
2024	[8,21,22,25,27,28,33,39,52,66,78,83,85,91,92,99,120,134,135,142,208,210,211,216,234,235,236,237] ***	9,4,7,0,3,3,5,0,8,18,7,2,0,7,3,0,5,3,19,9,13,12,19, 31,27,21,10,12,0	257
2025	[19,34,49,50,51,57,67,70,112,181,196,214] ***	1,10,0,2,8,2,2,2,0,0,0,5	33
Total citation			12,500

Notes: Publications on systematics and utility traits of plants of the genus *Magnolia* (*), anticancer activity of honokiol (**), main topic of the manuscript (***), methods of honokiol extraction extract (E).

## 12. Conclusions

Honokiol can be used in the treatment of viral skin diseases. It inhibits DNA replication (HSV-1, KSHV) through modulation of the IFN pathway, reduces gene expression and viral protein levels, regulates viral envelope glycoproteins and endosome colonisation, and restrains endocytosis. Honokiol derivatives containing a piperidine ring and its amphiphiles with high membrane selectivity, low cytotoxicity, and haemolytic activity are active against drug-resistant bacteria causing skin diseases, including *A. baumannii*, *P. gingivalis*, *P. intermedia*, *P. acnes*, *P. aeruginosa*, *S. aureus*, and *S. faecalis*, through disruption of biofilm formation and damage to cell walls and membranes. Honokiol is a safe fungicidal and fungistatic agent against dermatophytes that impairs ergosterol biosynthesis with squalene accumulation and induces cytosolic acidification by reduction of plasma membrane Pma1p H+-ATPase activity, leading to abnormalities in vacuole function and disruption of intracellular calcium homeostasis in the cells of dermatophytes from the genera *Aspergillus*, *Candida*, *Microsporum,* and *Trichophyton*. This compound inhibits fungal growth by delaying germination, altering membrane permeability, reducing hyphal elongation and branching, and disrupting biofilm formation at different stages via the Ras1-cAMP-Efg1 pathway.

Honokiol exerts anti-inflammatory effects via the SLC3A2/L-leucine/mTORC1/NLRP3 pathway and suppression of the TGF-β pathway. It reduces the level of inflammatory cytokines (IL-1β, IL-4, 6, 8, 13, 17, and IFN-γ) and the expression of pro-inflammatory NLRP3, COX-2, PGE2, and PCNA, stimulates potent anti-inflammatory IL-10, and enhances the expression of antioxidant activity-linked genes (SOD2) and HO−1 and Nrf2, lowering plasma nitrite levels. Honokiol has a protective function against ROS generation by restoration of mitochondrial function, improvement of senescence-associated phenotypes, and enhancement of skin barrier function.

Honokiol enhances matrix proteinases inhibitor TIMP-2 and reduces metabolically active SA-β-gal positive cells contributing to tissue damage and age-related diseases. It is a safe agent preventing UVB-induced skin cancer via targeting inflammatory mediators (lowering COX-2, PGE2, PCNA, TNF-α, IL-1β, and IL-6 levels), cell cycle regulators (CDK inhibitor upregulation), and cell survival signals (cyclins D1, D2, E2 and CDKs2, 4, 6 inhibition). It also induces mitochondrial (extrinsic) and death receptor (intrinsic) apoptosis pathways, activating proapoptotic proteins, including ‘inhibitor’ caspases-8 and -9, ‘executioner’ caspase-3, and caspase-mediated PARP cleavage. Honokiol inhibits the oncogenic KRT18 protein in melanoma and prevents recurrence of advanced melanoma using the β-catenin/MITF axis via prompt calpain-10 and CCAAT homologous protein (CHOP/GADD153) regulated cascades. Strategies for future clinical applications are necessary in order to benefit from the health-promoting potential of honokiol and its analogues in the supportive therapy of selected dermatological conditions.

## Figures and Tables

**Table 1:** Honokiol content in selected organs of various *Magnolia* species.

*Magnolia* Species	Organ		Honokiol Content (mg·g^−1^ D.W.)	Reference
*M. champaca*	bark		5.09	[52]
	flower		4.09	
*M. denudata*	bark		5.89	
	flower		2.10	
*M. grandiflora*	bark		6.61	
	flower		18.92	
*M. obovata*	leaf		0.70	[94]
	flower	petals	1.74	
		sexual parts	4.70	
*M. officinalis*	flower		13.20	[52]
	bark		56.79	
	trunk		0.08–25.28	[79]
	branches		6.64	
	root		87.56–96.51	
*M. officinalis* var. *biloba*	trunk		13.12–18.37	
	bark		3.18–18.37	[102]
	leaf		0.06–9.76	
*Magnolia* *× pruhoniciana*	leaf		0.11–8.26	[94]
	flower	petals	7.25–18.90	
		sexual parts	17.13–55.34	
*M. tripetala*	leaf		191.62	
	flower	petals	252.26	
		sexual parts	230.82	

**Table 2:** Physical and chemical properties of honokiol extracted from *Magnolia* plants.

Name/Parameter	Characteristics	Reference
IUPAC name	3′,5-Di(prop-2-en-1-yl)[1,1′-biphenyl]-2,4′-diol	[2,109]
Synonym name	3,5′-diallilo-4,2′-dihydroxybiphenyl	[112]
	4-allyl-2-(3-allyl-4-hydroxy-phenyl)phenol	
Molecular formula	C_18_H_18_O_2_	[2,89,113]
Physical state 20 °C	White crystalline solid	[114]
Molecular weight	266.334 g·mol	[3]
Affinity for water	hydrophobic	[16]
Melting point	86–86.5 °C	[109,114]
Boiling point	400 °C	[109]
Heat of evaporation	67.7 kJ·mol^−1^	[109]
Solubility	dimethyl sulfoxide: 36 mg·mL^−1^	[110,112,115]
	water: practically insoluble	
	methanol, ethanol: very soluble 33 mg·mL	
	soluble in chloroform, benzene, and caustic alkali	
Odour	spicy and fragrant	[109,116]

**Table 3:** Antiviral activity of honokiol in cell lines.

Honokiol/Extract/Other Chemical Compounds	Virus	Cell Lines	Honokiol Activity	Reference
Honokiol: 0, 10, 20 µM	DENV-2	BHK, Huh-7	Honokiol (HK) inhibited DENV-2 replication and endocytosis, as well as the expression of viral genes and NS1, NS3, and dsRNA proteins; it also disrupted the co-localisation of DENV envelope glycoproteins and early endosomes. Hence, honokiol may be used as a therapeutic agent in the chemotherapy of DENV infections.	[148]
Honokiol extract: 0, 5, 10, 15, 25, and 50 μg mL^−1^, acyclovir,	HSV-1, MHV68, KSHV	Vero, MEF	HK reduced the replication of the DNA of HSV-1, MHV68, and KSHV viruses and blocked the expression of HSV-1 viral genes at the levels of RNA and proteins; this activity increased when combined with acyclovir. HK may be a new ingredient in the treatment of HSV-1 and other herpes viruses.	[137]
Honokiol and α-mangostin at concentrations of 0, 5, and 10 µM)	MAYV	Vero-E6, HDF, HeLa	HK and α-mangostin shielded Vero-E6 cells against MAYV virus and reduced viral RNA replication in HeLa. They limited the infection with MAYV, Una, Chikungunya, and Zika viruses, inhibited the expression of viral proteins E1 and nsP1, and increased the expression of type I IFNs (IFN-α/β) and interferon-stimulated genes (*IFN α*, *IFN β*, *TNF α*, *ISG15*, *MxA*, *MDA-5*, *OAS2*, and *IL-1β*). Both these compounds exhibit wide-ranging anti-arbovirus activity involving different mechanisms.	[149]
Honokiol derivatives 3-((5-phenyl-1,3,4-oxadiazol-2-yl)methyl)oxazol-2(3 H)-ones	SARS-CoV-2	HEK-293-T-ACE2	HK derivatives were effective agents against SARS-CoV-2 as blockers of human ACE2 and were safe for host cells.	[150]
Honokiol thioethers containing 1,3,4-oxadiazole fragments			Some honokiol thioethers blocked SARS-CoV-2, binding to the ACE2 receptor via a specific immunological phenomenon involving T cell receptors called dual recognition of the SARS-CoV-2 RBD spike and human ACE2 and were not cytotoxic; hence, they may be inhibitors of α-D-glucoside glucohydrolase and SARS-CoV-2 entry.	[87]
Honokiol, magnolol, trans-3-Phenylacrylic (cinnamic) acid (concentration of each: 100 µM), furin inhibitor 25 µM		VeroE6	HK slowed down SARS-CoV-2 infection and an analogue site in SARS-CoV-2 S protein and limited SARS-CoV-2 infection via inhibition of furin-like activity and blocking PI3K/Akt signalling.	[151]
Honokiol: 0, 7, 8 μM		VeroE6, A549	HK prevented the SARS-CoV-2-mediated cytopathic effect (50% effective concentration 7.8 μM), reduced viral RNA copies and infectious progeny titres, and inhibited SARS-CoV-2 replication, including its variant Omicron, and other human coronaviruses at a post-entry step of the replication cycle via the expression of transmembrane protease serine 2 and angiotensin-converting enzyme 2. Honokiol is strongly recommended for animal and clinical studies to evaluate its effect on virus replication and pathogen–host (inflammatory) interplay.	[152]

Notes: A549—human basal alveolar epithelial cell lines, ACE2—angiotensin-converting enzyme 2, Akt—protein kinase B, BHK—Syrian hamster kidney cell lines, DENV-2—dengue virus serotypes, HDF—human dermal fibroblast cell lines, HEK-293-T-ACE2—ACE2-overexpressing human embryonic kidney 293 cells HEK-T cell lines, HeLa—cervical cancer cell lines, HK—honokiol, HSV-1—herpes simplex virus-1, Huh-7—epithelial tumour cell lines, IFNα—interferon alpha, IFNβ-interferon beta, IL-1β—interleukin-1 beta, ISG15 – interferon stimulated gene 15, KSHV—Kaposi’s sarcoma-associated herpes virus, MAYV—Mayaro virus, MDA-5—Melanoma Differentiation-Associated protein 5, MEF—mouse embryonic fibroblast cell lines, MxA—human myxovirus protein A induced by type IFNs (IFNα,β,ω) or by some viruses, MHV68—murine gammaherpesvirus 68, OAS2—2’, 5’-oligoadenylate synthetase 2, SARS-CoV-2—severe acute respiratory syndrome coronavirus 2, TNF α—tumour necrosis factor α, PI3K—Phosphoinositide 3-kinase, Vero E6 [VERO C1008, Vero 76, clone E6,] -African green monkey *Cercopithecus aethiops* kidney epithelial tissue cell lines.

**Table 4:** Antibacterial activity of honokiol in biological models.

Honokiol/Extract/Other Compounds	Bacterial Species/Strain	Minimal Inhibitory Concentration (MIC)	Main Conclusions	Reference
*in vitro*				
Honokiol, magnolol: 0, 50, 90%	Methicillin-resistant *Staphylococcus aureus* Methicillin-susceptible *S. aureus*	MIC/MBC at 16–64 mg·L^−1^ against the susceptible strain MIC_90_/MBC_90_ of 64/64 (magnolol) 32/64 mg·L^−1^ (honokiol) against the resistant strain	Honokiol (HK) showed similar activity to that of amikacin, gentamicin, and levofloxacin against the methicillin-susceptible *S. aureus* strain. It exhibited lesser anti-methicillin resistant potency than vancomycin and etilmicin but stronger efficacy than magnolol, gentamicin, amikacin, levofloxacin, piperacillin, fosfomicin, ciprofloxacin, or norfloxacin. Honokiol and magnolol exerted a synergistic effect, enhancing the activity of conventional antibiotics. The resistance reversal effects, especially those of amikacin and gentamicin against *S. aureus*, are related to the modulation of bacterial cell membrane penetration and cell wall damage.	[158]
Honokiol, magnolol: 10 mg·mL^−1^	Methicillin-resistant *S. aureus*	1 μg·mL^−1^	In murine phagocytes, honokiol and magnolol enhanced antioxidant activity in *S. aureus* infection by reducing ROS and inflammatory cytokines/chemokines, increased interferon type I and III mRNA expression, and inhibited *S. aureus* internalisation by human lung alveolar epithelial cells, which confirms the immunomodulatory and antimicrobial activity. Studies on the non-parasitic nematode *Caenorhabditis elegans* suggest antimicrobial activity of both neolignans from *Magnolia* bark *in vivo*.	[156]
*Lonicera japonica* ethanol extract, ethyl acetate fraction (50%), *Magnolia obovata,* ethanol extract, ethyl acetate fraction (70%)	*B. subtilis*, *E. coli*, *P. aeruginosa*, *S. aureus*	MIC (μg·mL^−1^) of magnolol and honokiol 31.2 (*S. aureus*) 10,000.0 (*E. coli*, *B. subtilis*) 1250.0 (*P. aeruginosa*)	*L. japonica* 50% ethyl alcohol extract was effective against *S. aureus* and *P. aeruginosa*, while *L. japonica* ethyl acetate fraction, as well as *M. obovata* 70% ethanol and ethyl extracts, were effective against all tested bacterial strains. This activity was related to the presence of neolignans (honokiol and magnolol) in *M. obovata* and polyphenols (luteolin and caffeic acid) in *L. japonica*. Synergistic antibacterial activity against *B. subtilis* and *S. aureus* was found using a combination of both ethyl acetate fractions, which suggests their potential use as natural preservatives in cosmetics.	[157]
Honokiol, magnolol (98%)	*Acetinobacter baumannii* ATCC 17978	500 μg·mL^−1^ (honokiol) 1000 μg·mL^−1^ (magnolol)	Honokiol and magnolol inhibited biofilm formation in five and four clinical isolates of *A. baumannii*, respectively. They reduced pellicle development and the gilding motility of this pathogen and extended lifespan of infected nematodes *Caenorhabditis elegans*; therefore, they may be useful in combating infections with this bacterium.	[159]
Honokiol and its derivatives	*S. aureus*, *S. pyogenes*, *S. albus*, *S. epidermidis*, *E. faecalis*, *E. coli*, *K. Pneumoniae*, *P. aeruginosa*	256 μg·mL^−1^ (*E. coli* and *P. aeruginosa*), 8–32 μg·mL^−1^ (*K. pneumoniae* and *S. pyogenes*), 16 μg·mL^−1^ (*S. aureus* and *S. epidermidis*), 16–32 μg·mL^−1^ (*S. faecalis*)	Among the 12 honokiol derivatives, the 7c derivative containing the piperidine ring showed a particularly broad spectrum of antibacterial efficacy, including monoderm and diderm species, which was more potent than that of linezolid, vancomycin, honokiol, and the other derivatives of this compound. This derivative probably destroyed the cell walls of the bacteria (*S. aureus* ATCC25923) at 1 × MIC and 4 × MIC.	[11]
Honokiol and magnolol (10, 20 and 50 µg·mL^−1^, magnolia bark methanol extract (30, 40, and 50 µg·mL^−1^. DMSO was used as the control	*Streptococcus mutans*	MIC of magnolia bark extract: 40 µg·mL^−1^, MIC of honokiol and magnolol: 10 µg·mL^−1^ in relation to chlorhexidine: 0.25 µg·mL^−1^	Honokiol exhibited time- and dose-dependent germ-killing activity against *S. mutans* planktonic cells and restrained the multi-step and complex biofilm formation process where free-floating bacteria attach to a surface, multiply, and create a protective matrix. Due to poor penetration, the biofilm is resistant to antibiotics. Magnolia bark extract exerted anti-biofilm action at concentrations higher than 30 µg·mL^−1^. Honokiol (50 30 µg·mL^−1^) and chlorhexidine (500 µg·mL^−1^) were characterised by lower penetration potential and bactericidal effects on *S*. *mutans* biofilm than magnolol (50 µg·mL^−1^) at 5 min post-exposure. All these agents exhibited scant biofilm-inhibiting activity after 30 s. In contrast to chlorhexidine, honokiol showed low toxicity towards gingival epithelial cells Ca9-22.	[160]
Honokiol and magnolol (0, 2.5, and 5 µg·mL^−1^)	Oral pathogens *A. actinomycetemcomitans*; *S. mutans*; *S. aureus*; methicillin-resistant *S. aureus* MRSA; *E. coli*	Honokiol MIC/MBC (µg·mL^−1^) *A. actinomycetemcomitans* 10/10; *S. mutans* 10/20; MRSA 10/20; *S. aureus* 10/90; *E. coli* > 100 > 100 Magnolol MIC/MBC (µg·mL^−1^) *A. actinomycetemcomitans* 10/20; *S. mutans* 10/20; MRSA 20/30; *S. aureus* 10/90; *E. coli* > 100 > 100	Honokiol decreased microbial biofilm formation and reduced pathogen antibiotic resistance. Honokiol at 5 µg·mL^−1^ repressed the *femA* and *femB* gene expression, essential for the formation of the cell wall peptidoglycan layer and methicillin resistance, and the expression of the *mecA* and *mecR1* genes. The mecA gene encodes the unique protein PBP2a with transpeptidase activity, which is not inhibited by β-lactam antibiotics (methacyclin), while the *mecR1* gene encodes the β-lactam-sensing transmembrane signalling protein MecR1. Magnolol had no impact on the *femA* and *femB* expression but upregulated the *mecR1* expression and repressed the *mecA* and *mecI* expression. The latter gene encodes a transcription repressor protein, MecI. The biofilm formation and expression of genes that contribute to antibiotic resistance were consistent with the respective phenotypes. Both honokiol and magnolol (2.5–10 μg·mL) efficiently repressed the expression of pro-inflammatory genes (*IL-*6 *IL-1β*, *COX-2*, and *iNOS* mRNA) in RAW264.7 cells. Honokiol and magnolol concentrations of 2.5–5 µg·mL^−1^ upregulated, while the dose of 10 µg·mL^−1^ downregulated, the *TNF-α* mRNA.	[161]
DMSO-dissolved honokiol or magnolol (200, 500, and 1000 μg·mL^−1^) and equivalent DMSO concentrations as the control	*A*. *baumannii* 17978 and other clinical *A. baumannii* isolates, antibiotic resistant ATCC BAA 1709 and A 571, as well as multidrug-resistant strains: A 550, A553, A 556, A 564, A 578, and A 580)	Not applicable	Both honokiol and magnolol efficiently disrupted biofilm formation dose-dependently and dispersed matured (established) *A. baumannii* ATCC 17978 biofilms via triggering ROS formation. Both neolignans greatly restricted biofilm formation by high biofilm formers ATCC BAA 1709 and A550 isolates, intermediate biofilm formers A564 and A580, and low biofilm-forming strain A571 but did not exert an anti-biofilm effect on two other less efficient biofilm formers, A553 and A556, and an intermediate biofilm former, A578. Honokiol and magnolol efficiently repressed the formation of a thin surface-attached layer (a pellicle) at a liquid–air interface, suppressed the surface motilities of *A. baumannii*, and extended *in vivo* the survival of infected nematodes *Caenorhabditis elegans*. Therefore, these major bioactive polyphenols of *Magnolia officinalis* may be useful for preventing the spread and managing existing *A. baumannii* infections.	[159]
Honokiol (0.5, 1, 2, 4, and 8 μg·mL^−1^)	*S. aureus* ATCC 29213 and 9 clinical *S. aureus* isolates (SA 56, SA 121, SA 1987, SA 1862, SA 2985, SA 3015, SA 3101, SA 3303, and SA 3629)	MIC values against the tested strains in the suspension ranging within 4–16 μg·mL^−1^ MBC values ranging within 8-32 μg·mL^−1^	*S. aureus* isolates produce various types of biofilms mediated by the main component of the bacterial biofilm extracellular matrix (extracellular DNA, eDNA) or partially deacetylated N-acetylglucosamine polymer polysaccharide intercellular adhesion (PIA) with high importance for cell-to-cell adhesion and biofilm accumulation produced by the icaADBC operon. Honokiol was recognised as an efficient agent against *S. aureus* biofilm cultures, as its MBICs for strains grown in the biofilm cultures oscillated between 32 and 128 μg·mL^−1^. Honokiol detaches mature biofilms, destroys bacteria in biofilms, prevents mRNA production from genes *icaA*, *cidA*, and *sarA*, and therefore inhibits eDNA release by *S. aureus* and downregulates PIA expression. Gene *icaA,* encoding N-acetylglucosaminyl-transferase, is essential for PIA synthesis and regulates the synthesis of exopolysaccharide matrix components. Gene *CidA,* related to cell lysis and eDNA release, enhances extracellular murein hydrolase activity. Staphylococcal-specific SarA gene family transcription regulators control large numbers of target genes involved in, e.g., biofilm formation. The *SarA* gene plays a pivotal role in biofilm formation capacity, acting as a regulator for virulence factors and biofilm development.	[162]
Honokiol and magnolol dissolved in 10% DMSO	*Propionibacterium acnes* ATCC 6919 and *Propionibacterium granulosum* ATCC 25564	MIC against both tested strains: honokiol 3–4 μg·mL^−1^ (11.3–15 μM), magnolol 9 μg·mL^−1^ (33.8 μM)	Honokiol and magnolol may be regarded as promising acne-alleviating candidates for cutaneous application without causing any adverse reactions. Both neolignans killed *P. acnes* swiftly, with 10^5^ organisms/mL eliminated within 10 min upon either honokiol (20 μg·mL^−1^; 75.2 μM) or magnolol (45 μg·mL^−1^; 169.2 μM) treatment. Honokiol showed higher cytotoxicity than magnolol in human normal fibroblasts and HaCaT cell lines, but both these neolignans exhibited similar cytotoxicity in co-administration with an acne-mitigating agent triclosan. Honokiol and magnolol (concentrations of 5, 10, and 20 μM) lowered the secretion of *P. acnes*-induced pro-inflammatory cytokines (TNF-α, IL-8) in human leukaemia monocytic cell line (THP-1) cells.	[153]
*in vivo*				
Amphiphiles of honokiol and magnolol: 0,2 mM; 53.3 mg	Methicillin-resistant *S. aureus*	0.5–2.0 μg·mL^−1^	Among various new honokiol/magnolol amphipaths that mimic the molecular structures and germ-killing properties of cationic amphipathic peptides, compound 5i, with low cytotoxicity (CC_50_ > 128 μg·mL^−1^), haemolytic activities (HC_50_ = 789.2 μg·mL^−1^), and high membrane selectivity (SI = 789.2), showed strong activity against low-drug-resistance *S. aureus* isolates. 5i disrupts biofilm and damages bacterial cell membranes, has strong anti-infective efficacy in a mouse model of sepsis (female BALB/c mice) with *S. aureus* isolate, and may be a safe antibacterial agent in the therapy of bacterial infections.	[150]
0.3% Water-ethanol extract from *M. grandiflora* bark	*S. mutans*	0.3 mg·mL^−1^	Clinical studies conducted on a group of volunteers (18–35 years old) not using any mouthwash or antibiotics showed that *Magnolia* bark extract used as mouthwash had an antibacterial effect against *S. mutans* in dental plaque, reduced the concentration of *S. mutans* on teeth, and decreased the volume of microbial plaque on dental surfaces and the plaque index.	[163]

Notes: COX-2—cyclooxygenase 2, DMSO—dimethyl sulfoxide, HaCaT cell line—spontaneously immortalised human keratinocyte cell line, IL-1β—interleukin-1 beta or leukocytic pyrogen, IL-6—interleukin-6, IL-8—interleukin-8, iNOS—inducible nitric oxide synthase, MIC—minimal inhibitory concentration, MBC—minimum bactericidal concentration, MBICs—minimum biofilm inhibitory concentrations, PBP2a—penicillin-binding protein 2a, ROS—reactive oxygen species, TNF-α—tumour necrosis factor α.

**Table 5:** Antifungal activity of honokiol in biological models.

Honokiol/Extract/Other Compounds	Fungi/Strains	MIC/MFC Minimum Fungicidal Concentration	Main Conclusions	Reference
*in vitro*				
*Magnolia obovata*, ethanol extract, ethyl acetate fraction (70% each), *Lonicera japonica* ethanol extract, ethyl acetate fraction (50% each)	*A. brasiliensis*—mould *C. albicans*—yeast	70% ethyl acetate fraction from *M. obovata* MIC (μg·mL^−1^): 625 (*C. albicans*), 2500 (*A. brasilensis*);—70% ethanol extract MIC (μg·mL^−1^): 1250 (*C. albicans*), 1000 (*A. brasilensis*); magnolol and honokiol MIC (μg·mL^−1^): 5000 (*C. albicans*), 39 (*A. brasilensis*)	Both the 70% alcoholic and ethyl acetate *M. obovata* extracts exhibited microbicidal activity against *A. brasilensis* and *C. albicans*. A synergistic effect against *C. albicans* was shown for the combination of two ethyl acetate fractions. Magnolol and honokiol were responsible for the antibacterial activity in *M. obovata*. These two neolignans exhibited strong activity against *A. brasilensis*. The combination of extracts can be a promising natural plant preservative.	[157]
Honokiol 16 μM	*C. albicans*	16 μg·mL^−1^	Honokiol (HK) inhibited the biosynthesis of ergosterol (a steroid playing a key role in fungal cell membranes), blocked the activity of plasma membrane proton-ATPase (PM H+-ATPase or Pma1p), thus inducing cytosolic acidification of the cytosol, caused abnormalities in vacuole function and morphology, and disrupted intracellular calcium homeostasis. Amiodarone-induced calcium influx lessened honokiol toxicity against *C. albicans* via stimulation of the calcineurin signalling pathway participating in honokiol tolerance.	[168]
Honokiol and magnolol (8, 16, 32, and 64 μg·mL^−1^)	Clinical isolates of *C*. *albicans* strains (SC5314, CA1, CA2, CA3, CA4, CA10, CA127, CA129, CA132, CA135, and CA137)	MICs 16–32 μg·mL^−1^	Honokiol restricted adhesion to the HSC-T6 cells and free-floating state of *C. albicans* cells in a liquid medium (planktonic growth), protected against yeast-to-hypha transition, and inhibited biofilm growth at the early, developmental, and biofilm maturation stages via the Ras1-cAMP-Efg1 pathway. This critical regulator of *C. albicans* biofilms is activated by extracellular environmental factors that stimulate the Ras1 protein, which in turn induces adenylate cyclase (Cyr1) to synthesise the second messenger cAMP from ATP. Honokiol and magnolol (16 μg·mL^−1^*) in vivo* extended the lifespan of *C. albicans*-infected nematodes *Caenorhabditis elegans*.	[59]
*ex vivo; in vivo*				
Honokiol, magnolol: 0, 12.5, 25, 50 µM	*Trichophyton ajelloi, T. gypseum, T. mentagrophyte, T. rubrum, Microsporum canis*	MIC 8 mg·mL^−1^ (honokiol, magnolol) MFC 16 mg·mL^−1^ (honokiol, magnolol)	Both neolignans had strong anti-dermatophyte activity as a result of ergosterol biosynthesis suppression with simultaneous enhancement of squalene accumulation in fungal cells due to limitation of squalene conversion into 2,3-oxidosqualene. They did not have cytotoxic effects on human neutrophils in an *ex vivo* assay but influenced the generation of lipopolysaccharide (LPS)-stimulated cytokines in human neutrophils: magnolol inhibited the secretion of IL-1β, IL-8, and TNF, while honokiol inhibited IL-1β. Synergy was found for the magnolol–terbinafine combinations (FICI = 0.50), while the mixtures of HK with terbinafine (positive control) displayed an additive effect (FICI = 0.56). HK and magnolol, which act via impairment of ergosterol biosynthesis, can be effective fungicidal agents against dermatophytes.	[7]
Honokiol: 0, 2, 4, 8, 16 μg·mL^˗1^. Honokiol concentrations no more than 8 µg/mL were considered nontoxic and were used in the experiments	*A. fumigatus*	MFC 12 μg·mL^−1^	Honokiol exhibited fungistatic (at 6 h) and fungicidal action (at 24 h). HK inhibited *A. fumigatus* growth, lessened its adhesion ability, diminished biofilm formation, and increased membrane permeability. In an *in vivo A. fumigatus* keratitis murine model (8-week-old females), HK reduced fungal loading, diminished the number and vitality of neutrophils, and repressed TLR-2 action, inflammatory cytokines (TNF-α, IL-1β), and amphoterin, also known as HMGB1. HK may be a powerful antimycotic and anti-inflammatory agent for *A. fumigatus* keratitis.	[164]
Honokiol (5 μL) and mannan (10 mg·mL^˗1^), Dectin-2siRNA (10 μM) or honokiol (8 μg/mL) three times a day (9:00 a.m., 1:00 p.m., 5:00 p.m.)	*A. fumigatus*	MIC 8 µg·mL^−1^	Compared to the honokiol-supplemented group, 383 downregulated and 1175 upregulated genes were activated in C57 black 6 mice keratomycosis with PBS treatment. Proteins encoded by these genes are involved in fungal defence and immune activation. HK inhibited fungal growth through delaying germination and alterations in membrane permeability *in vitro* and reducing hyphal load *in vivo*. The antiphlogistic HK action was based on reduced expression of multiprotein complex NLRP3 releasing IL-1β as well as the pattern recognition receptor for fungal Dectin-2 that initiates inflammatory responses and signal transmitting in innate immune cells. HK may be effective and safe in mitigation of fungal keratitis symptoms.	[165]

Notes: cAMP—cyclic adenosine monophosphate, FICI—fractional inhibitory concentration index, HK—honokiol, HSC-T6 Rat Hepatic Stellate cell line, HMGB1—high mobility group box 1, IL-8—interleukin 8, IL-1β—interleukin-1 beta, MIC—minimal inhibitory concentration, MFC—minimal fungicidal concentration, NLRP3—NLR family pyrin domain-containing 3 inflammasome, LPS—lipopolysaccharide, TLR-2—toll-like receptor-2, PBS—phosphate-buffered saline, TNF—tumour necrosis factor.

**Table 6:** Anti-inflammatory activity of honokiol in biological models (*in vitro*).

Honokiol	Research Model	Experimental Groups (G)	Duration	Main Conclusions	Reference
Honokiol	Human foreskin fibroblasts [HFF-1; Culture Collection Type SCRC-1041] and human keratinocyte cell line (HaCat).	G1 and G2—two control groups of HFF-1 and HaCat cell lines. G3 and G4—cigarette-smoke-exposed HFF-1 and HaCat cultures. Cultures of human fibroblasts and keratinocytes incubated with two non-cytotoxic HK concentrations, 10 and 20 μM, and exposed to cigarette smoke.	48 h	Honokiol (HK) was a strong and safe anti-inflammatory, anticollagenolytic, anti-apoptotic, and anti-senescence agent against cigarette smoke. In cell cultures exposed to tobacco smoke, HK decreased IL-8 and IL-1α production in keratinocytes, prevented degradation of fibroblast-produced collagen I and IV via enhancement of the activity of matrix proteinases inhibitor TIMP-2, shielded fibroblasts from apoptosis, and diminished β-galactosidase expression in the fibroblasts. HK may be recommended for basic science and clinical use.	[9]
Honokiol dissolved in 0.1% DMSO	Primary cultures of normal buccal mucosal fibroblasts (BMFs) known as normal cheek fibroblasts 2. Patient-derived fibrotic buccal mucosal fibroblasts (fBMFs) derived from precancerous oral submucous fibrosis (OSF) tissues.	1G: control; 2G: arecoline; 3G: arecoline + 5 μM HK; 4G: arecoline + 10 μM HK; 5G: arecoline + 20 μM HK. Normal BMFs seeded at 1 × 10^4^ cells/well and fBMFs treated with HK for 48 h (5 replicates for each HK concentration).	48 h	HK is a promising safe agent inhibiting oral fibrogenesis progression and preventing oral mucosa transformation into cancer. HK suppressed arecoline-induced myofibroblast activities, e.g., collagen gel shrinkage, cellular motility, and wound repair capacities. The anti-fibrotic effect of HK resulted from arecoline-elicited improvement of myofibroblast activities via TGF-β pathway suppression. Increasing HK concentrations in BMFs suppressed the arecoline-induced TGF-β secretion. HK inhibited arecoline-stimulated Smad2 phosphorylation together with α-1 type I collagen and α-SMA downregulation. HK attenuated tubulointerstitial fibrosis by restraining the ECM and inflammatory mediators.	[23]
Honokiol	Normal human epidermal keratinocytes (NHEKs).	1G: control 100 µM 2-aminoethoxydiphenyl borate (2-ABP); 2G: 2-ABP 100 µM + 10 µM HK; 3G: 2-ABP 100 µM + 30 µM HK; 4G: 2-ABP 100 µM + magnolol 10 µM; 5G: 2-ABP 100 µM + magnolol 30 µM; 6G: DMSO; 7G: HK 10 µM; 8G: magnolol 10 µM.	24–48 h	Honokiol and magnolol strongly inhibited TRPV3 ion channels produced by keratinocytes and mitigated the TRPV3-channel agonists (carvacol) or GOF mutation-induced cytotoxicity. HK and magnolol obstructed the TRPV3 current (I_TRPV3_) and the rise in cytoplasmic calcium in NHEKs and HEK293T cells—derived from HEK cells—overexpressing hTRPV3 and its GOF mutants (G573S and G573C) linked with Olmstead syndrome (mutilating palmoplantar keratoderma). HK and magnolol suppressed the TRPV3 agonist-stimulated release of inflammatory cytokines (IL-6 and IL-8) from keratinocytes and may be potentially used to treat inflammatory skin diseases.	[21]
Honokiol	A human monocytic leukaemia cell line (THP-1) originating from the peripheral blood of an acute monocytic leukaemia (AML-M5) patient was cultured on RPMI 1640 medium with supplementation of 10% FBS. Differentiated THP-1 cells were obtained via culturing two types of cells together with p-methoxyamphetamine (100 ng·ml^−1^ PMA for twenty-four hours). Then, BMDMs were cultured in DMEM with 10% FBS and 30% fibroblast-like cell line derived from mouse tissue (L929) culture supernatants were obtained from C57BL/6 mouse femora and shinbones.	In studies on HK-suppressed NLRP3 inflammasome priming and activation processes, HK was applied for 40 min to LPS-primed and ATP (5 mM)-stimulated THP-1 cells for an hour. In order to probe possible HK targets, the LPS-primed THP-1-cell lysates were incubated for 45 min with HK at concentrations of 10, 100, and 1000 μM. Pronase (Actinase E) was administered for additional 30 min for hydrolysis. In experiments concerning HK-promoted SLC3A2 degradation via the proteasome pathway, HK was applied to LPS-treated and then ATP-stimulated THP-1 cells. Prior to the LPS exposure, the cells were treated for an hour with either 5 mM autophagy blocker 3-MA or 10 µM strong proteasome blocker MG132. The LPS-stimulated THP-1 cells were additionally energised or not by ATP in the absence or presence of HK.	7 days	Honokiol exerted severe anti-inflammatory effects *in vitro.* It considerably diminished IL-1β release and caspase-1 (interleukin-1 converting enzyme) activation at various time points and therefore suppressed the key component of the nonspecific (innate) immune system, the Nod-like receptor pyrin domain-containing 3 (NLRP3) inflammasome priming, and inhibited cell activation processes.	[171]

Notes: BMDMs—bone marrow-derived macrophages; DMEM—Dulbecco’s Modified Eagle Medium, ECM—extracellular matrix, FBS—foetal bovine serum, GOF—gain-of-function, HaCat—immortalised human keratinocytes, HEK—human embryonic kidney cells, HFF-1—human foreskin fibroblasts IL-6—interleukin-6, IL-8—interleukin-8, IL-1β—interleukin 1β, 3-MA—3-methyladenine, TRPV3—transient receptor potential vanilloid-3, α-SMA—alpha-smooth muscle actin, TIMP-2—tissue inhibitors of metalloproteinases-2, TGF-β—Transforming Growth Factor-beta.

**Table 7:** Anti-inflammatory activity of honokiol in biological models (*ex vivo*).

Honokiol	Research Model	Experimental Groups (G)	Duration	Main Conclusions	Reference
Honokiol 18 mg	Human neutrophil model	G1: LPS-stimulated (10 ng·mL^−1^) human neutrophils (LPS+); G2: negative control cells (LPS–); G4–6: HK 12.5, 25, and 50 μM in LPS-stimulated neutrophils; G7–9: magnolol 12.5, 25, and 50 μM in LPS-stimulated neutrophils. Both neolignans were applied 30 min prior to neutrofil LPS-stimulation.	24 h	Honokiol (HK) and magnolol as dual activity therapeutic agents, i.e., with selective fungistatic and anti-inflammatory activity, can be used to modulate the balance between anti- and pro-inflammatory signals in human host–tinea interactions. Their anti-inflammatory properties support tissue repair and lesion regeneration process and alleviate ringworm symptoms. The tested concentrations of both neolignans did not induce cytotoxicity in polymorphonuclear leukocytes, compared to the LPS-negative control. HK and magnolol affected the e*x vivo* LPS–cytokine production in human neutrophils: HK decreased IL-1β, while magnolol showed an inhibitory effect towards IL-8, IL-1β, and TNF-α.	[7]
Honokiol	Microglia (Hortega cells) and astroglia (star shaped cells) dissected from midbrain and cerebral hemispheres of 2-day-old Sprague Dawley or Wistar rats	Control: unstimulated cells. Treatments: HK between 1 μM and 100 μM.	48 h	Honokiol markedly reduced the LPS-induced inflammatory response of astroglia and microglia primary cultures via the inhibition of inflammatory molecules (IL-1β, IL-6, iNOS, and TNF-α), as well as anti-inflammatory cytokine (IL-10) stimulation. HK mitigated the LPS-induced expression of epithelial zinc finger protein, also known as zinc finger transcription factor KLF4, in microgliocytes and astroglia. HK shows great potential in early research for further development as a drug candidate for the treatment of neuroinflammatory disorders.	[172]
Honokiol	Foetal (extraembryonic) membranes, tunica muscularis (myometrium or muscular coat), freshly isolated amniotic epithelial cells, and myocytes (primary myometrial cells or uterine smooth muscle cells)	1G: control; 2G: lipopolysaccharide (LPS); 3G: LPS + 100 µM HKu; 4G: fibroblast-stimulating lipopeptide-1 (fsl-1); 5G: fsl-1 + 100µM HK; 6G: 20 µg·ml^−1^ poly(I:C) (poly(I) • poly(C))—immunostimulant polyinosinic–polycytidylic acid; 7G: poly(I:C) +100µM HKu	G1, 2, 4, 6 60 min G. 3, 5, 7 20 h -	Honokiol severely reduced the inflammation-triggering substance (IL6, IL1A) and chemotactic cytokine (CCL2, CXCL1, CXCL8) mRNA expression and secretion from extraembryonic membranes (amnion and the amnion-surrounding layer choriodecidua) and the muscular middle layer called myometrium stimulated with poly(I:C), LPS, or fsl-1. In the amniotic epithelial cells, HK diminished the expression and secretion of the extracellular enzyme gelatinase B (matrix degrading enzyme MMP9). In the myometrium, HK severely inhibited the contraction-associated protein PTGFR expression, as well as the secretion of uterotonic prostaglandins PGE_2_ and PGF_2α_, but blocked TNF-induced uterine muscle contraction. In primary amnion and the muscular middle layer of the uterine wall (myometrial) cells, HK suppressed the pro-inflammatory cytokine IL1B- and TNF-induced transcriptional activity of NF-κB RelA, which is a strong transcriptional activator of pro-inflammatory genes. HK reduced the expression of pro-labour and inflammatory mediators in human amnion, choriodecidua, and tunica mascularis through suppressed activation of NF-κB. HK was recognised as a strong therapeutic agent preventing preterm birth.	[173]

Notes: HK—honokiol, IL-6—interleukin 6, IL-8—interleukin 8, IL-1*α*—interleukin-1 alpha, IL-10—anti-inflammatory cytokine interleukin 10, IL-1β—interleukin 1β, iNOS—inducible nitric oxide synthase, KLF4—Krüppel-like factor 4, MMP9—matrix metalloproteinase 9, NF-κB—nuclear factor κB, PGE_2_—prostaglandin E2, PGF_2α_—prostaglandin F2α, PTGFR—prostaglandin F receptor, TNF-α—tumour necrosis factor α.

**Table 8:** Anti-inflammatory activity of honokiol in biological models (*in vivo*).

Honokiol	Research Model	Experimental Groups (G)	Duration	Main Conclusions	Reference
Honokiol (a specific neolignan type) dissolved in 2% DMSO and physiological saline given before carrageenan- or CFA-induced inflammation	adult male 20–30 g BALB/c mice	I. experiment: carrageenan-induced (100 µL/paw) biological inflammation model groups: 1G: normal—saline with 2% dimethyl sulfoxide (DMSO); 2G: carrageenan; 3G: piroxicam (10 mg·kg^−1^ i.p.); 4G–6G: carrageenan + HK 0.1, 5, and 10 mg·kg^−1^ (i.p.). Carrageenan and piroxicam were administered intraperitoneally 4 h post carrageenan (100 µL/paw) administration. II. experiment: Freund’s complete adjuvant (FCA-induced skin inflammation) groups: 1G: vehicle control; 2G: CFA (20 µL/paw) injected into the back foot plantar surface; 3G: dexamethasone Dexa 5 and 10 mg·kg^−1^ (i.p.), 4G. HK 10 mg·kg^−1^ (i.p.)	I experiment 4 h II experiment 0–6 days	Honokiol (HK) exerted anti-allodynic and anti-hyperalgesic effects in sharp and persistent pain models induced by CFA and carrageenan and attenuated pain induced by thermal hyperalgesia. HK reduced the expression of CFA- and carrageenan-induced inflammatory cytokine mRNA (IL-1β, IL-6, TNF-α, and VEGF—a signal protein involved in angiogenesis). HK upregulated genes linked to antioxidants (SOD2) and enhanced HO-1 and Nrf2 expression, while reducing plasma nitrite levels. Studies with HK agonists [gabapentin (5 mg·kg^−1^), piroxicam (5 mg·kg^−1^), tramadol hydrochloride (50 mg·kg^−1^) i.p.] and antagonists [flumazenil (0.2 mg·kg^−1^), naloxone (4 mg·kg^−1^) and olanzapine (10 mg·kg^−1^), i.p.] revealed that HK exerted analgesic activity via inhibition of anti-inflammatory mediators. Muscle activity and liver function tests revealed that HK is reasonably safe and may be a promising candidate for ongoing development as an intervention option for chronic and acute pain.	[174]
Honokiol	7-week-old Bagg Albino BALB/c mice (males)	1G: PBS-treated controls (phosphate-buffered saline (PBS) (pH 7.4); 2G: 1-chloro-2, 4-dinitrobenzene (DNCB); 3G: HK (5 mg·kg^−1^) + DNCB; 4G: HK (10 mg·kg^−1^) + DNCB; 5G: dexamethasone (DEX 10 mg·kg^−1^) + DNCB.	24 h	Honokiol (10 mg·kg^−1^) administration suppressed DNCB-induced mastocyte accumulation and inflammation. HK reversed DNCB-induced enhancement in serum immunoglobulin E levels. HK ameliorated the DNCB-induced rise in pro-inflammatory cytokines (interferon-γ, IL-4, 13 and 17) in the skin and lymph glands. Simultaneously, HK reversed the DNCB-induced enlargement in the lymph node size. HK (3,5′-diallyl-4,2′-dihydroxybiphenyl) markedly reduced DNCB-induced atopic reactions in the ears and lymph glands. HK may be a prospective therapeutic agent in eczema treatment.	[3]
Honokiol	6-week-old SKH-1 hairless mice (females)	I. Time-effect experiments with a topical low dose of HK application before and after UVB exposure on tumour incidence groups: 1G: control acetone; 2.G: HK 2 h before UVB exposure; 3G: HK 1 h before UVB exposure; 4G: HK 30 min before UVB exposure; 5G: HK immediately after UVB exposure; 6G: HK 30 min after UVB exposure; 7G: HK 30 min after UVB exposure. Topical HK treatment—30 μg of HK in 200 μL of acetone UVB exposure 30 mJ·cm^−2^. II. Experiments concerning the effects of pretreatment with HK on tumour incidence in UVB-induced skin carcinogenesis groups: 1G: control (200 μL of acetone); 2G: HK 30 μg; 3G: HK 45 μg; 4G: HK 60 μg. Topical HK treatment: applications of 30, 45, and 60 μg of HK in 200 μL of acetone, respectively. HK was administered one hour before UVB exposure (30 mJ/cm^2^, Monday–Friday).	27 weeks—induction of cancer in mouse cells (experiment I); 25 weeks—HK treatment (experiment II)	In the time-reaction and dose-response experiments, HK severely supressed skin tumour multiplicity by 49–58% and 36–78%, respectively, and diminished tumour volumes by 70–89% and 76–94% in a dose-related manner. Moreover, HK dose 60 μg diminished tumour incidence by 40%. Low HK doses (30 μg), when applied either prior to or after UVB rays, exhibited cancer-preventive effects. HK arrested UVB-induced skin cancer in a dose-related manner. HK was recognised as a safe and effective chemoprotective agent against skin cancer. The study of skin tumours revealed that HK induced apoptosis via extrinsic and intrinsic pathways, impeded UVB-induced inflammation and inflammatory factors, lowered cell survival cues and proliferation markers, and upregulated cell cycle inhibitor proteins, but downregulated cell cycle promoter proteins.	[175]
Honokiol in 0.5% CMC-Na	Specific pathogen free (SPF) grade, 22–25 g adult male ICR mice	1G, Control, 2G. LPS (1 mg·kg^−1^, i.p.), 3G. LPS + HK (10 mg ·kg^−1^, per os). Each experimental group consisted of 10 individuals.	11 days	Honokiol inhibited the inflammatory responses showing antidepressant effects. This compound ameliorated the LPS-induced NF-κB activation in the hippocampus and lowered the peripheral blood levels of inflammatory cytokines (TNF-α, IL-1β, and brain IFN-γ). It affected tryptophan metabolism, decreasing IDO expression, reduced the level of toxic quinolinic acid, reduced brain tissue free calcium, in consequence inhibiting calcium overload, and increased neuroprotective metabolites.	[104]
Honokiol	8-week-old 20–22 g C57BL/6 mice (males)	I. LPS-induced septic shock groups: 1G, LPS (20 mg·kg^−1^ i.p.) alone, 2G. LPS +HK (35 mg/kg per os p.o.), 3G, LPS + HK (70 mg·kg^−1^ per os). II. Dextran sulphate sodium (DSS)-induced colitis “prevention model”) groups: 1G, The vehicle group fed regular water received the vehicle once a day per os; 2G, Model group fed with dextran sulphate sodium (DSS) water received vehicle once a day per os; 3G and 4G. HK (35 and 70 mg·kg^−1^) groups fed DSS water and received HK (35 or 70 mg·kg^−1^ p.o. daily). III. “therapy model” groups: 1G, Vehicle-administered group (p.o. once a day) drinking regular water; 2G, Model group fed DSS water for a week; 3G. and 4G. HK (35 and 70 mg·kg^−1^) groups pre-drinking DSS water for a week and next receiving HK (35 or 70 mg·kg^−1^ daily p.o.) for 5 days; 5G, Positive group pre-drinking DSS water for a week receiving 5-aminosalicylic acid (100 mg·kg^−1^ daily p.o.) for 5 days	40 min	Honokiol exerted severe anti-inflammatory efficacy *in vivo*. HK mitigated LPS-induced inflammation, a severe, life-threatening stage of sepsis, and DSS-induced colon inflammation *in vivo* (“prevention and therapy model”) and suppressed adaptor apoptosis speck-like protein oligomerisation. The SLC3A2 protein coding gene was identified as a direct target of HK in the THP-1 cells. HK directly bound to SLC3A2 and downregulated its expression via enhanced proteasome-mediated proteolysis. HK triggered SLC3A2 to suppress cryopyrin (NLRP3) inflammasome activation via lowering L-leucine content transported into cells and lysosomes to block the mTORC1 pathway. Therefore, HK was recognised as a potential anti-inflammatory agent acting via the SLC3A2/L-leucine/mTORC1/NLRP3 pathway.	[171]

Notes: CMC-Na—sodium carboxymethylcellulose, DMSO—dimethyl sulfoxide, DNCB—dinitrochlorobenzene, DSS—dextran sulphate sodium, FCA—Freund’s complete adjuvant or complete Freund’s adjuvant (CFA), HK—honokiol, HO-1 haem oxygenase-1, IDO—indoleamine 2,3-dioxygenase, IFN-γ interferon γ, IL-1β—interleukin 1β, IL-4—interleukin-4, IL-6—interleukin 6, IL-13—interleukin 13, IL-17—interleukin 17, LPS—lipopolysaccharide, mTORC1—Mammalian Target of Rapamycin Complex 1, Nrf2—nuclear factor erythroid 2-related factor 2, NF-κB—nuclear factor kappa B, NHEKs—normal human epidermal keratinocytes, NLRP3—nod-like receptor pyrin domain-containing 3 inflammasome, PBS—phosphate-buffered saline, SLC3A2—solute Carrier Family 3 Member 2, SOD2—superoxide dismutase 2, SPF—specific pathogen free, THP-1 cells—monocyte cell line derived from the whole blood of an acute monocytic leukaemia patient, TNF-α—tumour necrosis factor α, VEGF—vascular endothelial growth factor.

**Table 9:** Photoprotective activity of honokiol extracted from *Magnolia* plants in selected biological models.

Honokiol /Extract	Research Model	Experimental Groups (G)	Duration	Main Conclusions	References
*in vitro*					
*M. officinalis* extract. Bark mixed with 70% ethanol in a volume ratio of 1:8 was heated at 60° C for 3 h.	Cell lines: 1G: immortalised human keratinocytes (HaCaT); 2G: human dermal fibroblasts (PCS–201–010); 3G: normal human epidermal keratinocytes (HEKn). Senescent fibroblasts with a doubling time of 14 days and young fibroblast (positive control) with a doubling time of less than 2 days.	1G: *M. officinalis* extract (0.625 and 10 μg·mL^−1^) with a strong antioxidant resveratrol (100 µM)—a positive control and DMSO control (0.001%); 2G: honokiol (0.1; 1, and 10 μM) with *M. officinalis* extract (0.625 μg·mL^−1^) as a positive control and DMSO control (0.001%).	Extract (0.625 or 10 μg·mL^−1^) was administered to senescent fibroblasts at the specified concentrations for 12 days. For measurement of skin moisture retention, keratinocytes were exposed to DMSO (0.01%) or *M. officinalis* extract (0.625, 1.25, and 2.5 µg·mL^−1^) for 72 h	Honokiol (HK) is the major anti-oxidation agent in *M. officinalis* extracts. The non-toxic doses of this neolignan 1 and 10 μM reduced mitochondrial ROS generation more efficiently than the *M. officinalis* extract dose of 0.625 μg·mL^−1^. The HK treatment of aged fibroblasts promoted a cell proliferation comparable to the *M. officinalis* extract. HK reduced mitochondrial ROS generation through mitochondrial functional recovery due to a rise in MMP, resulting in lower mitochondrial mass, lower basal levels of glycolysis, and reduced post 2–DG acidification, suggesting a less intense residual glycolysis process that was not blocked by 2–DG. HK, similar to the *M. officinalis* extract, reduced the dependence on glycolysis as an energy source, indicating restoration of mitochondrial function by HK. Honokiol functioned as an oxygen radical scavenger via disproportionation reactions, limited ROS levels due to revitalisation of mitochondrial activity, and consequently improved senescence-associated phenotypes and enhanced the skin barrier function. HK reduced the SA–β–gal positive cells, lowered intracellular lipofuscin levels, increased SLIT2 expression, indicating honokiol-mediated enhancement of skin regeneration, inhibited the expression of MMP1 responsible for collagen degradation, decreased the expression of CCL2 and 5, and lowered IL-6 and 8, thereby mitigating skin inflammation.	[181]
*in vivo*					
Honokiol (30 μg in 0.2 mL acetone)	Highly susceptible to UVR-induced skin cancer SKH-1 hairless five-week-old female mice	Two groups of mice: G1: control (200 μL of acetone topically); G2: HK 30 μg in 200 μL of acetone administered topically 1 h before UVB exposure-initiated carcinogenesis (30 mJ/cm^2^/day) 5 days/week for 30 weeks.	30 weeks	Honokiol did not affect weight gain; it maintained normal growth and development of mice and resulted in a reduction in tumour multiplicity and a decrease in the mean ratio of tumour area to total back area. Mechanistic studies revealed the proapoptotic potential of honokiol as a consequence of DNA fragmentation induction through the extrinsic (death receptor) pathway, in which the binding ligand–cell surface receptor activates caspases 8 and 3 and the intrinsic (mitochondrial) pathway via up-regulation of caspases 9 and 3, PARP, and tumour suppressor p53. This is crucial for maintaining homeostasis and determines the anti-photocarcinogenic action, making honokiol a strong topical chemopreventive, anti-skin-cancer agent.	[179]
Honokiol in a hydrophilic cream topical preparation	Highly susceptible to UVR-induced skin cancer SKH-1 hairless five-week-old mice	Three groups of mice with 7 individuals per group. 1G: control; 2G: UVB (180 mJ/cm^2^) treatment; 3G: honokiol applied externally prior or subsequent to irradiation.	48 h	Honokiol administered topically prior to and after UVB irradiation led to (1) enhanced photoprotection in the context of tumour multiplicity and volume per tumour-transplanted mouse, (2) suppressed and reduced malignant progression of non-cancerous papillary tumour to carcinomas, (3) inhibited UVB-stimulated gene expression of PGE2, COX-2, PCNA, and inflammation-promoting molecules (TNF-α, IL-1β, IL-6) (4) decreased levels of cyclins (D1, D2, E2) and associated CDK2, CDK4 and CDK6, (5) upregulated cyclin-dependent kinase inhibitor Cip/p21, also known as p21 WAF1 or Kip/p21, crucial for cell cycle arrest, (6) suppressed PI3K activity crucial in cellular signalling and inhibited Akt at Ser473 phosphorylation responsible for Akt activation in UVB radiation-induced skin tumours. HK is a promising agent for the photoprotection through targeting inflammatory mediators, cell cycle control molecules, and cell survival signals in UVB-irradiated skin.	[178]
Hydrophilic ointment with honokiol (0.5 and 1.0 mg/cm^2^ skin area). The hydrophilic ointment base served as a vehicle for this topical formulation.	Female 5–6-week-old C3H/HeN mice homozygous for the Pde6brd1 allele. Normal—compared to wild type littermates with any gross phenotypic differences, COX-2 knockout (+/−) mice reproductive pairs kept within the 129 Ola/C57BL/6 mixed genetic background.	Five groups of mice. 1G: normal control; 2G: HK alone; 3G: UVB radiation (150 mJ/cm^2^) for four consecutive days); 4G: HK (0.5 mg/cm^2^ of skin area) + UVB; 5G: HK (1.0 mg/cm^2^ of skin surface) + UVB. HK was applied to shaved mouse skin in a hydrophilic cream-based topical formulation starting 3 days prior to the start of the UVB exposure and thereafter at 30 min preceding each exposure to 2 and 4 mg honokiol (4% and 8% (*w*/*w*) in the topical preparation) per mouse/50 mg vehicle.	24 h	Honokiol prevented UVB-induced suppression of the allergic contact dermatitis response via the COX-2 synthesised principal potent lipid inflammatory mediator PGE_2_ but did not restrict UVB-induced cutaneous hypersensitivity suppression in Ptgs2-/- (COX-2 knockout) mice. Honokiol inhibited UVB-induced DNA hypermethylation, stimulated TET enzyme (methylcytosine dioxygenase) activity responsible for DNA dimethylation, and elevated the activity of large (~180- to 230-kDa) TET multidomain proteins in UVB-irradiated mouse skin. The DNA hypomethylating agent nucleoside analogue decitabine (5-aza-2′- deoxycytidine) and the potent COX-2 inhibitor nonsteroidal anti-inflammatory drug indomethacin enhance the contact hypersensitivity response in UVB-exposed mice. Comparative studies of the effects of topical application and gavage feeding of honokiol (2 mg per mouse; equal to 100 mg·kg^−1^ body weight) on UVB-mediated immunosuppression in a murine model of allergic contact dermatitis revealed quite similar inhibition of contact hypersensitivity between the two administration routes of this neolignan. The local application of equal concentrations (18.8 mM) of HK and two widely available drugs (imiquimod and 5-fluorouracil) used in the topical chemotherapy or immunotherapy did not significantly affect the UVB-mediated immunosuppression.	[10]

Notes: Akt—serine/threonine protein kinase B, CDK2, CDK4, and CDK6—cyclin-dependent kinase 2, 4, and 6, COX-2—cyclooxygenase-2 or prostaglandin-endoperoxide synthase, DMSO—dimethyl sulfoxide, HK—honokiol, IL-1β—interleukin-1 beta, IL-6—interleukin-6-type, MMP—mitochondrial membrane potential, PARP—poly(ADP-ribose) polymerase, PCNA—proliferating cell nuclear antigen, PGE2—prostaglandin E2, SLIT2—slit guidance ligand 2, TET—ten-eleven translocation proteins, TNF-α—tumour necrosis factor-α.

**Table 10:** Anti-melanoma activity of honokiol in biological models (*in vitro*, *in vivo*).

Honokiol	Research Model	Experimental Groups (G)	Duration	Main Conclusions	Reference
*in vitro*					
Honokiol	Human melanoma cell lines: G1—A375 cells with epithelial morphology isolated from the skin of a 54-year-old cutaneous melanoma female patient; G2—Hs294t cell line established from a human melanoma metastasis to the lymph node; groups 3 and 4—SK-Mel119 and SK-Mel28 melanocytes from the skin of a 51-year-old malignant melanoma male patient with unspecified ethnic origin.	Honokiol-treated melanoma cell lines treated (0, 5, 10, and 20 μM for 42 h)	24 h	Honokiol (HK)-treated metastatic melanoma-derived cell line cultures revealed a dose-dependent limitation of cell migration due to Nox1 downregulation and relieved oxidative stress. Diphenyleneiodonium chloride, a Nox1 inhibitor, decreased the migration of SK-Mel28 and Hs294t cells with high and low expression, respectively, of SIRT3, a protein serving as a treatment target of both tumour-suppressive and oncogenic function. Honokiol enhanced the build-up of the *NCF1* gene-encoded cytosolic p47phox protein but diminished the membrane-bound p22phox protein level, thus completely preventing their interaction and blocking Nox1 activation in the SK-Mel28 and Hs294t cells.	[223]
Honokiol	Melanoma cell lines: group 1—SKMEL-2 with polygonal morphology from the skin of a 60-year-old cutaneous melanoma white male patient; group 2—UACC-62 derived from a 41-year-old male with pancreatic ductal adenocarcinoma metastatic to the peritoneal mass.	Honokiol-treated SKMEL-2 and UACC-62 cells (0, 25, 50, 70, and 100 μM for 12, 24, 48, and 72 h)	12–72 h	Honokiol (1) reduced cell viability, cell multiplication, and cell cycle arrest but enhanced programmed cell death and modulation of cell cycle regulatory and apoptotic proteins in both cell lines; (2) induced cell cycle quiescence in UACC-62 and G2/M checkpoint arrest in SKMEL-2 cells; (3) elevated the level of caspases and PARP responsible for maintaining viability in the cell lines but lowered the expression of cell cycle regulatory proteins.	[221]
Honokiol	Malignant melanoma cell lines: G1—B16F10 originating from C57BL/6J mice; G2—A375 with epithelial morphology from the skin of a 54-year-old malignant melanoma female patient; G3—MeWo with fibroblast morphology from the skin of a 78-year-old white malignant melanoma male patient; G4—A2058 with epithelial morphology from the skin of a 43-year-old white metastatic melanoma male patient.	Honokiol-treated malignant melanoma cells (0–100 μM)	24–92 h	Honokiol suppressed the growth and metastasis of melanoma. This neolignan caused ER stress CHOP activation and lowered β-catenin, CDK2, and MITF expression in mouse melanoma tissues, thus increasing the survival likelihood. Honokiol effectively prevented the progression of advanced melanoma using the Wnt/β-catenin-MITF pathway (β-catenin/MITF axis or β-catenin-dependent MITF regulation pathway) via prompt calcium-dependent non-lysosomal cysteine protease CAPN10) and CCAAT-enhancer-binding protein (C/EBP) homologous protein (CHOP/GADD153) regulated cascades.	[121]
Honokiol	Human melanoma cell lines: G1—SK-MEL-5 (with high metastatic potential, more sensitive to some chemotherapeutics than SK-MEL-28; used for invasion and migration tests; G2—SK-MEL-28 (used in studies on targeted therapy; lines with moderate resistance to apoptosis with BRAF V600E mutation with glutamic acid at amino acid instead of valine at position 600).	Honokiol-treated (0, 10, 20, 30, and 40 µM) SK-MEL-5 and SK-MEL-28 cell lines	24–96 h	Honokiol effectively suppressed the growth (anchorage-independent growth, cell proliferation, colony forming ability) of melanoma cells in a dose- and time-varying manner. Honokiol interacted directly with a key for malignant status type I cytokeratin KRT18 protein and induced its ubiquitin-mediated degradation. Increase in the level of KRT18 expression promoted melanoma cell multiplication and growth.	[73]
*in vivo*					
Honokiol	4–6-week-old athymic hairless female mice as a tumour cell invasion model	Three groups of mice. Honokiol administration: 1G—100 mg·kg^−1^ body weight supplemented with 0.5% carmellose dissolved in 200 μL of sterilised water/mouse; 2G—vehicle administered via oral gavage for 7 weeks 5x per week. 3G—melanoma A375 cells (2.5 × 10^6^) *i.v.* injected intravenously into the tail vein of mice either not provided with or orally supplied with honokiol.		Honokiol prevented the migration/extravasation and growth of intravenously injected melanoma cells in the liver, kidney, and lung of athymic mice as a consequence of a suppressing effect on Nox1 activity in the above-mentioned organs.	[223]
Honokiol	Five–six-week-old heterozygous Nu+ thymus-less, thus unable to produce T-cells, immunodeficient hairless (nude) male mice NU/NU (NU-FOXN1(NU)). They have no rejection response, can receive multiple tissue types and tumour grafts, and are widely used for tumour biology and xenograft research.	Two groups of mice. 1G—receiving a nontoxic honokiol dose of 50 mg·kg^−1^ dissolved in gingelly oil (i.p.); 2G—control group, injected with an equal volume of gingelly oil (i.p.).	Animal treatment for 2–7 weeks three times per week	Honokiol potently accelerated tumour regression in UACC-62 and SKMEL-2 melanoma xenografts in the athymic mice. It is recommended to be further studied as an effective supportive therapy for malignant melanoma.	[221]
Honokiol	Severe combined immunodeficient (SCID) CB-17 mice with recessive autosomal mutation characterised by an absence of functional T and B cells, lymphopaenia, hypogammaglobulinaemia, and an unchanged hematopoietic environment (normal NK cells, macrophages, and granulocytes).	Groups receiving honokiol at 30–140 mg·kg^−1^ oral or i.p.	Six weeks of treatment three times per week	Honokiol prevented UVB-induced skin cancer via targeting chemical mediators of inflammation (lowering PGE2, COX-2, PCNA, IL-1β, IL-6, and TNF-α levels), cell cycle regulators (increased expression the CDK inhibitor), and cell survival signals (suppression of cyclins D1, D2, E2 and CDKs2, CDK4, and CDK6). It induced programmed cell death via death receptor (extrinsic) and mitochondrial (intrinsic) pathways, activating proapoptotic proteins, including upregulation of caspases 8, 9, and 3 and enzymatic PARP breakdown (PARP cleavage)—an apoptosis marker that facilitates cellular disassembly through DNA shearing and suppressing DNA repair. Honokiol inhibited the melanoma cell growth promoter KRT18 and prevented advanced melanoma progression using the β-catenin-dependent MITF regulation pathway via prompt calpain-10 and CCAAT-enhancer-binding proteins (C/EBPs) homologous protein (CHOP/GADD153) transcription factor-regulated cascades.	[121]
Honokiol	SK-MEL-5 or SK-MEL-28 melanoma cell-derived xenograft mouse models. Five–six-week-old severe combined immunodeficient, i.e., “living in the bubble” syndrome or “bubble boy disease,” female CB 17 mice.	Three G of mice. 1G—vehicle control (1% carmellose sodium in physiological saline), 2G—honokiol 30 mg·kg^−1^, and 3G—50 mg·kg^−1^ (i.p.).	20 days	Honokiol decreased KRT18 expression and suppressed the growth of melanoma cell lines implanted into immunodeficient mice (cell derived xenograft models). It reduced neoplasia marker Ki-67 expression. Honokiol acts as an inhibitor of the oncogenic protein KRT18 in melanoma.	[73]

Notes: CAPN10—calpain-10, CDK—cyclin-dependent kinase, C/EBP—CCAAT-enhancer-binding protein, CHOP—pro-apoptotic transcription factor C/EBP homologous protein, DNA damage-inducible transcript 3 or growth arrest- and DNA damage-inducible gene 153 (*GADD153*), ER—endoplasmic reticulum, KRT18—keratin 18 protein, Nox1—NADPH oxidase 1, p47phox protein—neutrophil cytosol factor 1 protein, *NCF1* gene—neutrophil cytosolic factor 1 gene, NK cells—natural killer, MITF—microphthalmia-associated transcription factor, p22phox protein—human neutrophil cytochrome blight chain (CYBA) or α-subunit of the flavocytochrome b558, i.p.—intraperitoneal injection, i.v.—intravenous injection, PARP—poly(ADP-ribose) polymerase, SIRT3—sirutin 3 mitochondria NAD+-dependent deacetylase.
